# A novel micellular fluorogenic substrate for quantitating the activity of 1-phosphatidylinositol 4,5-bisphosphate phosphodiesterase gamma (PLCγ) enzymes

**DOI:** 10.1371/journal.pone.0299541

**Published:** 2024-03-29

**Authors:** Ramya Visvanathan, Tadanobu Utsuki, Daniel E. Beck, W. Brent Clayton, Emma Lendy, Kuai-lin Sun, Yinghui Liu, Kirk W. Hering, Andrew Mesecar, Zhong-Yin Zhang, Karson S. Putt

**Affiliations:** 1 Institute for Drug Discovery, Purdue University, West Lafayette, IN, United States of America; 2 IUSM-Purdue TREAT-AD Center, West Lafayette, IN, United States of America; 3 Department of Medicinal Chemistry and Molecular Pharmacology, Purdue University, West Lafayette, IN, United States of America; 4 Division of Clinical Pharmacology, Indiana University School of Medicine, Indianapolis, IN, United States of America; 5 Department of Biochemistry, Purdue University, West Lafayette, IN, United States of America; 6 Cayman Chemical Company, Ann Arbor, MI, United States of America; Sungkyunkwan University and Samsung Advanced Institute of Health Science and Technology (SAIHST), REPUBLIC OF KOREA

## Abstract

The activities of the phospholipase C gamma (PLCγ) 1 and 2 enzymes are essential for numerous cellular processes. Unsurprisingly, dysregulation of PLCγ1 or PLCγ2 activity is associated with multiple maladies including immune disorders, cancers, and neurodegenerative diseases. Therefore, the modulation of either of these two enzymes has been suggested as a therapeutic strategy to combat these diseases. To aid in the discovery of PLCγ family enzyme modulators that could be developed into therapeutic agents, we have synthesized a high-throughput screening-amenable micellular fluorogenic substrate called C16CF3-coumarin. Herein, the ability of PLCγ1 and PLCγ2 to enzymatically process C16CF3-coumarin was confirmed, the micellular assay conditions were optimized, and the kinetics of the reaction were determined. A proof-of-principle pilot screen of the Library of Pharmacologically Active Compounds 1280 (LOPAC_1280_) was performed. This new substrate allows for an additional screening methodology to identify modulators of the PLCγ family of enzymes.

## Introduction

The phospholipase C (PLC) family of enzymes comprises thirteen members divided into six families designated as β, γ, δ, ε, ζ and η [1,2]. The PLC gamma (PLCγ) family comprises two isozymes, PLCγ1 and PLCγ2. PLCγ1 is ubiquitously expressed, while PLCγ2 is primarily expressed in immune cells and microglia with sparse expression in other brain cells such as astrocytes and oligodendrocytes [3]. Both isozymes, like all PLC enzymes, convert membrane PIP_2_ (phosphatidylinositol 4,5-bisphosphate, PtdIns(4,5)P_2_, PI(4,5)P_2_) into IP_3_ (1D-Myo-inositol 1,4,5-trisphosphate) and diacylglycerol (DAG). The formation of these secondary signaling molecules regulates numerous signaling pathways such as nuclear factor-κB (NF-κB), extracellular signal-related kinase, mitogen-activated protein kinase, and nuclear factor of activated T cells signaling pathways, which in turn control diverse cell functions such as cellular proliferation, endocytosis, and calcium influx [4]. Unsurprisingly, the dysfunction of PLCγ enzymes results in a variety of diseases, including immune disorders, cancer, and neurodegeneration [4,5].

Several activating mutations in PLCγ family enzymes cause immune dysregulation disease, auto-inflammation, and/or PLCγ2-associated antibody deficiency & immune dysregulation (APLAID) syndrome in humans [5,6], and induced inflammatory arthritis in mouse models [7]. Additional activating mutations in PLCγ family enzymes, although generally with higher rates of aberrant activity, also have been found in several cancers. For example, recurrent PLCγ1 mutants such as Ser345Phe have been identified in T-cell lymphomas and leukemias [8,9]. In B cell leukemia patients treated with ibrutinib, the PLCγ2 activating Arg665Trp and Leu845Phe mutations frequently arise which cause cancer recurrence [10,11]. Overactive PLCγ2 plays a role in diffuse large B-cell lymphomas as well [12]. Additionally, overexpression of PLCγ1 has been identified in gliomas and is associated with poor survival [13]. Thus, PLCγ1 and PLCγ2 inhibitors may be efficacious therapeutics for these diseases. Unfortunately, no selective, potent PLCγ1 or PLCγ2 small molecule inhibitors are currently known.

Therapeutics that selectively activate PLCγ1 and PLCγ2 could be useful in certain cancer and neurodegenerative indications as well. For example, an activating PLCγ2 mutant was protective in mice with gastric MALT lymphoma that was induced by the bacteria, *Helicobacter felis* [14]. Recently, Alzheimer’s disease patients with a rare mutation in PLCγ2 (i.e., Pro522Arg) were identified and patients with this activating mutation [3] exhibited a slower rate of cognitive decline [15–18]. Like inhibitors, no selective, potent activators of either of these two enzymes are known.

Clearly, modulators of PLCγ1 and PLCγ2 activity could be highly beneficial for a number of diseases. However, HTS-amenable assays must be developed before large-scale screening can be implemented to identify these modulators. Previously, PLC activity was measured using radiolabeled PIP_2_ [19]. However, as labs have moved away from the use of radioactivity-based assays, high-throughput-amenable optical assays were developed. Initially, these consisted of complex methods such as one utilized by the DuPont Merck Pharmaceutical company that 1) incubated PLC enzymes with phosphatidylinositol, 2) halted that PLC reaction via the addition of EDTA, 3) used alkaline phosphatase to hydrolyze the product of the PLC reaction to form inorganic phosphate and inositol, 4) halted that reaction via the addition of SDS and additional EDTA, 5) added ZnCl_2_ and ammonium molybdate to form a colored complex with the generated inorganic phosphate, and then 6) measured the absorbance to ultimately determine the activity of the original PLC enzyme [20].

Fortunately, far more facile methods have been developed more recently that utilize fluorescent reporter substrates, such as WH-15 [21], C8CF3-coumarin [22], and XY-69 [23]. WH-15 is a water-soluble substrate that is used in solution assays with PLC enzymes [21]. WH-15 underwent an extensive “turn-on” fluorophore optimization that resulted in the creation of C8CF3-coumarin [22]. The C8CF3-coumarin substrate maintains water solubility but greatly increases the amount of fluorescence intensity upon cleavage by PLC enzymes [22]. XY-69 is incorporated into a liposome and is currently considered the best assay for monitoring PLC enzymatic activity, because the substrate is only processed when the PLC enzyme is in contact with the membrane, thus mimicking the cellular environment [23]. The liposomal XY-69 assay is therefore more suitable to screen for allosteric inhibitors and of course can identify orthosteric inhibitors as well [24]. Unfortunately, suitable liposomes are tedious to prepare in large quantities and often suffer from high batch-to-batch variability. While the water-soluble substrates, WH-15 and C8CF3-coumarin, are far easier to utilize for screening applications, they are generally used to screen only for active site modulators [25].

In an effort to develop an assay that is as easy to implement as the solution assays, while also having the potential to identify allosteric modulators like the liposomal assay, a micelle-based PLCγ screening assay was developed. To accomplish this, a modified version of the solution assay substrate C8CF3-coumarin was synthesized (C16CF3-coumarin). As detailed herein, the enzymatic activity of PLCγ family enzymes upon this substrate was determined, the micellular assay was optimized, and a pilot screen of the LOPAC_1280_ library with PLCγ2 was conducted.

## Materials and methods

### Materials

Chemicals for synthesis were procured from well-known suppliers such as ThermoFisher Scientific (Waltham, MA, USA) and MilliporeSigma (Burlington, Massachusetts, USA). XY-69 was purchased from Avanti Polar Lipids (Alabaster, Alabama, USA). All of the chemicals used for the assay (HEPES, calcium chloride, potassium chloride, sodium chloride, dithiothreitol, glycerol, sodium cholate, and adenosine triphosphate) and consumables (pipette tips, microtiter plates) were procured from ThermoFisher Scientific (Waltham, MA, USA). Bovine serum albumin, EGTA, sodium cholate, and the LOPAC_1280_ library were purchased from MilliporeSigma (Burlington, MA, USA). The reagent stock solutions were prepared with water and stored at 4 or -20°C, according to the manufacturer’s instructions.

### Synthesis of C16CF3-coumarin

**Scheme 1:**
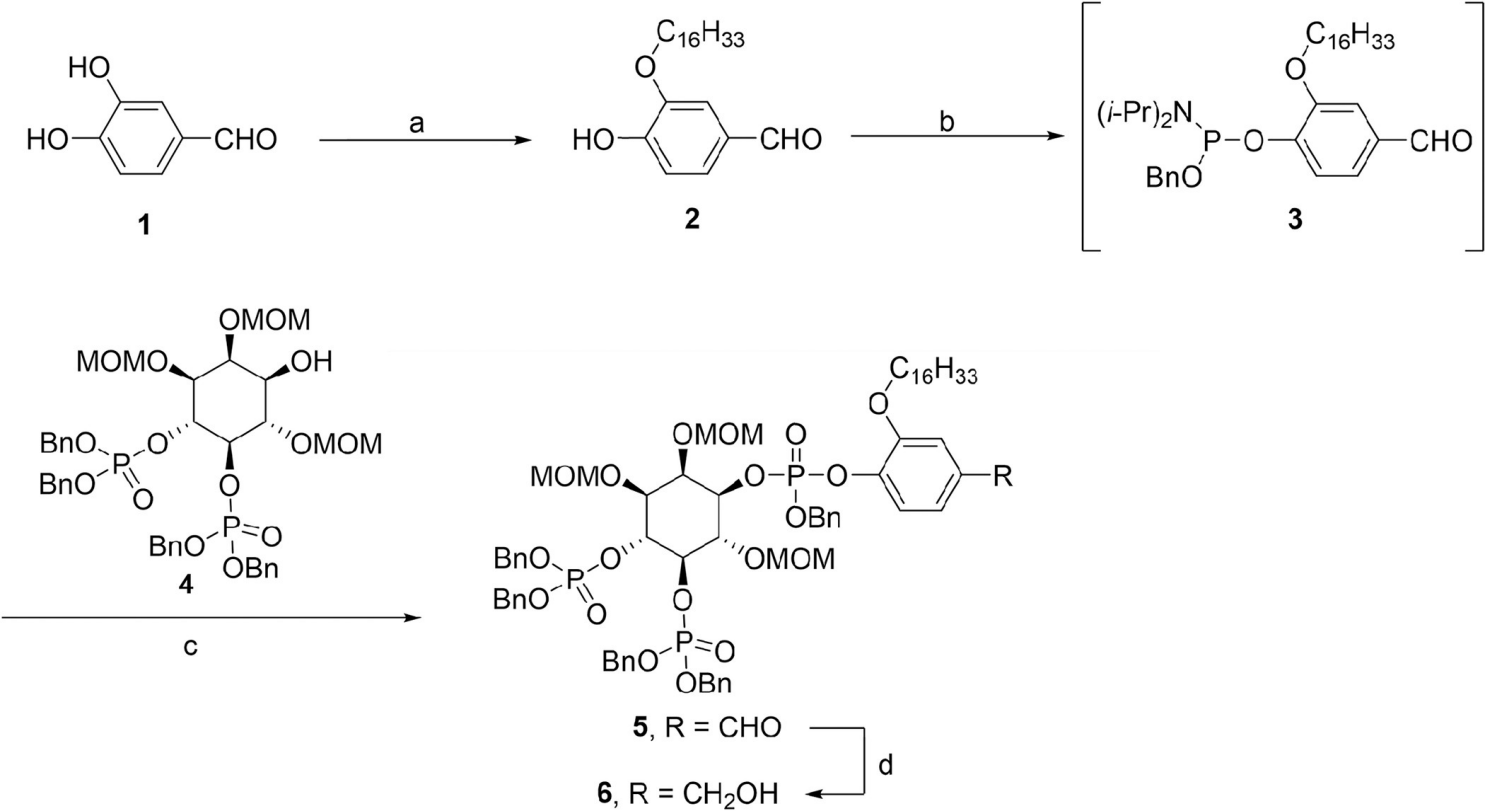
Reagents and conditions: (a) 1-bromohexadecane, NaH, DMF, THF, 0°C to rt to 50°C, 38%; (b) 1*H*-tetrazole, BnOP(N(i-Pr)_2_)_2_, ACN, THF, 0°C to rt; (c) i. 1*H*-tetrazole, THF, rt to 30°C to 35°C; ii. t-BuOOH, H_2_O, 0°C to rt, 46% (2 steps); (d) NaBH_4_, THF, rt, 72%.

**Scheme 2:**
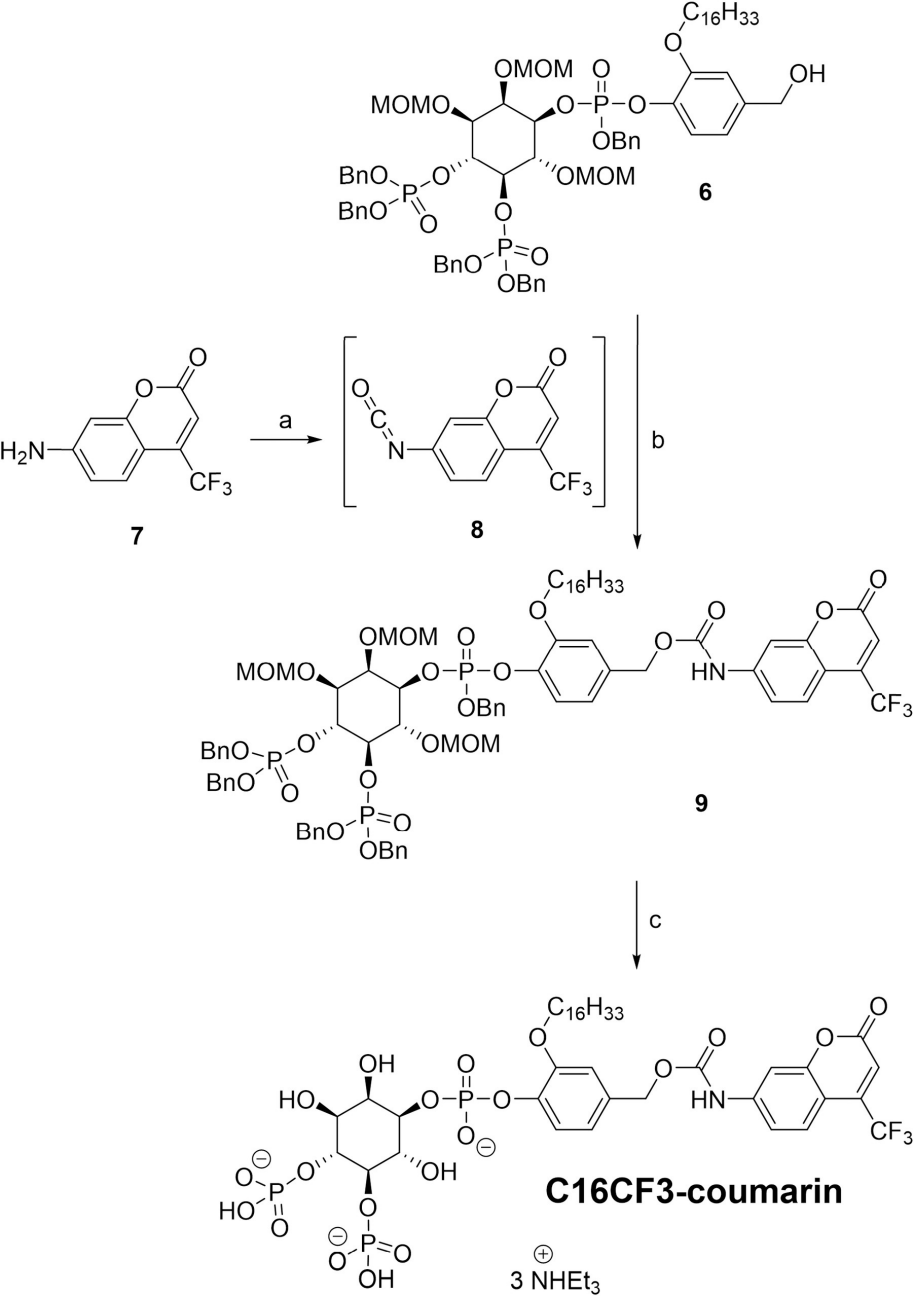
Reagents and conditions: (a) triphosgene, NEt_3_, DCE, 90°C; (b) NEt_3_, DMAP, 4 Å molecular sieves, DCM, rt, 96%; (c) i. TMSBr, DCM, 5°C to rt; ii. MeOH, rt; iii. aq TEAB, rt, 70%.

The synthesis of C16CF3-coumarin was accomplished following the routes shown in **Schemes 1** and **2**, which are similar to the published routes to related PLC substrates C8CF3-coumarin [22] and WH-15 [21]. Following 3-position selective alkylation of 3,4-dihydroxybenzaldehyde (**1**) [26] with 1-bromohexadecane to provide intermediate **2**, the latter was converted to phosphoramidite **3** by a standard protocol (**Scheme 1**). Intermediate **3** was condensed with the known protected inositol **4** in the presence of catalytic 1*H*-tetrazole, and the formed phosphite was oxidized to phosphoester **5** using *t*-BuOOH. It was found that the latter two transformations (i.e., **2** → **3** and **3** + **4** → **5**) could be most efficiently executed in a one-pot procedure, as described in the Supporting Information. Next, the aldehyde functional group of **5** was reduced using NaBH_4_ to provide advanced intermediate **6**. Fluorescent reporter 7-amino-4-trifluoromethyl-coumarin (**7**) was converted to isocyanate **8**, which was then coupled to the benzylic alcohol **6** to give carbamate **9** (**Scheme 2**). Global deprotection of **9** was executed using TMSBr, followed by sequential treatment with MeOH and then aqueous TEAB buffer. Flash RP LC purification on C8-derivatized silica gel provided the pure substrate C16CF3-coumarin in tris•NEt_3_ salt form, as determined by ^1^H NMR.

### Preparation of PLCγ2 and assay reagents

The PLCγ enzymes PLCγ2-WT, PLCγ2-P522R, and PLCγ1-WT were prepared as described in Visvanathan et al. [22]. PLCγ1-D1125H was a generous gift from the Sondek group at the University of North Carolina. The PLCγ enzymes were stored in a PLC storage buffer containing 20 mM HEPES (pH 7.4), 150 mM NaCl, 5% (v/v) glycerol, and 1 mM DTT at -80°C. C16CF3-coumarin (1 mM) was dissolved in water and stored at -80°C. Optimal concentrations of PLCγ enzymes and C16CF3-coumarin were diluted with micelle assay buffer [HEPES assay buffer containing 50 mM HEPES (pH 7.4), 3 mM EGTA, 70 mM KCl, 3 mM CaCl_2_, 2 mM DTT, 0.04 mg/ml of BSA (fatty acid-free), and 0.5% (w/v) sodium cholate].

### Optimization of assay conditions

To determine the optimal concentration of sodium cholate for the C16CF3-coumarin assay, PLCγ2 and C16CF3-coumarin were added to the HEPES assay buffer with 0–1% (w/v) sodium cholate. 10 μl of PLCγ2 (5 nM) and 10 μl of C16CF3-coumarin (10 μM) were dispensed into 384 well-black plates. The final assay concentrations were PLCγ2 (2.5 nM) and C16CF3-coumarin (5 μM). The assay plate was loaded on a Synergy-neo2 plate reader (Agilent, Santa Clara, CA, USA), and the fluorescence was monitored (excitation 362 nm; emission 496 nm) at 25°C every 2 min for 3 h. The slope was analyzed from the initial linear range (60 min) of reaction curves.

To determine optimal calcium chloride concentrations, C16CF3-coumarin (10 μM) and PLCγ2 (5 nM) were diluted with assay buffer containing 50 mM HEPES (pH 7.4), 3 mM EGTA, 70 mM KCl, 2 mM DTT, 0.04 mg/ml of BSA (fatty acid-free) and 0.5% (w/v) sodium cholate and 0–15 mM of calcium chloride. PLCγ2 (10 μl) and C16CF3-coumarin (10 μl) were dispensed into a microtiter plate, and fluorescence intensity was recorded as mentioned above. The final concentrations of PLCγ2 and C16CF3-coumarin were 2.5 nM and 5 μM, respectively. The slope was analyzed from the initial linear range (60 min) of reaction curves.

To find optimal concentrations of enzyme, substrate, and BSA, PLCγ2 (0–40 nM) and C16CF3-coumarin (5–40 μM) were diluted with assay buffer containing 50 mM HEPES (pH 7.4), 3 mM EGTA, 70 mM KCl, 3 mM CaCl_2_, 2 mM DTT, 0.5% (w/v) sodium cholate, and 0.02–0.08 mg/ml of BSA (fatty acid-free). PLCγ2 (10 μl) and C16CF3-coumarin (10 μl) were plated into a microtiter plate, and the fluorescence intensity was recorded as mentioned above. The final concentrations of PLCγ2 and C16CF3-coumarin were 0–20 nM and 2.5–20 μM, respectively. The slope was analyzed from the initial linear range (60 min) of reaction curves.

### Micelle distribution by Dynamic light scattering (DLS)

Dynamic light scattering measurements were made using a Malvern Zetasizer Pro instrument from Malvern Panalytical (Worcester, UK). All samples were prepared in deionized water (Millipore Milli-Q, 18.0 MΩ ⋅ cm^−1^). DLS measurements were taken 3 times, for 60 runs at 1.64 s run^-1^, with a 120 s equilibration time and an equilibration temperature of 25°C, per the instrument manufacturer’s instructions.

### PLCγ2 assay with C16CF3-coumarin

For the PLCγ2 assay with C16CF3-coumarin, mixtures of 0–40 nM of PLCγ2 and 10 μM of C16CF3-coumarin were prepared with micelle assay buffer. PLCγ2 (10 μl) and C16CF3-coumarin (10 μl) were dispensed into the microtiter plate, and the final concentrations were 0–20 nM and 5 μM, respectively. The fluorescence intensities from the assay reactions were monitored, as mentioned above. The slope was analyzed from the initial linear range (60 min) of reaction curves.

### Validation of PLCγ2 activity with C16CF3-coumarin

The PLCγ activity with C16CF3-coumarin was tested with PLCγ mutants and ATP, a known PLCγ inhibitor [23]. 1 μl of a 100 mM ATP solution was incubated with 10 μl of PLCγ2 WT and P522R enzymes for 15 min at RT. The assay reaction was commenced by adding 10 μl of C16CF3-coumarin. The final concentrations of assay reactions were ATP (4.8 mM), PLCγ2 (2.4 nM), and C16CF3-coumarin (4.8 μM). A similar procedure was followed for testing PLCγ1 and D1165H enzymes. The slope was analyzed from the initial linear range (60 min) of reaction curves.

### Determinations of kinetics parameters

For kinetic studies, 20 nM of PLCγ2 and 2–50 μM of C16CF3-coumarin were prepared with micelle assay buffer. 10 μl each of the PLCγ2 and C16CF3-coumarin solution were dispensed into the microtiter plate, and fluorescence intensity was monitored as described above. The final concentrations of PLCγ2 and C16CF3-coumarin were 10 nM and 1–25 μM, respectively. The slope was analyzed from the initial linear range (60 min) of reaction curves. For PLCγ1 kinetics with C16CF3-coumarin, a similar procedure was performed as mentioned above with final concentrations of PLCγ1 (2 nM) and C16CF3-coumarin (1–32 μM).

### Solution and liposomal assays

The solution assay using the C8CF3-coumarin substrate and the liposomal assay using XY-69 were performed as previously described [22].

### High throughput screen of the LOPAC_1280_

To obtain the Z’ factor for the assay with C16CF3-coumarin, PLCγ2 (0, 5, and 10 nM) and C16CF3-coumarin (10 μM) were prepared with micelle assay buffer. The final concentration of PLCγ2 (5 and 0 nM) served as controls for activation (2x activation) and inhibition in the assay with C16CF3-coumarin. 10 μl per well of prepared enzyme and 10 μl per well of substrate were dispensed into the microtiter plate with priming volume of 1 ml using a Mantis liquid dispenser (Formulatrix, Bedford, MA, USA). The final enzyme and substrate concentrations were 0, 2.5, and 5 nM for PLCγ2 and 5 μM C16CF3-coumarin. The enzymatic reactions were monitored by detecting their fluorescence, and the slope was analyzed from the initial linear range (60 min) of reaction curves.

When screening the compounds of the LOPAC_1280_ library_,_ an Echo 650 liquid dispenser (Beckman Coulter, Indianapolis, IN) was used to dispense 10 mM of compounds in DMSO (100 nl) or only DMSO. PLCγ2 (10 μl) and C16CF3-coumarin (10 μl) in micelle assay buffer were dispensed in the wells described above. The final concentration of PLCγ2 (0 and 5 nM) served as controls for inhibition and activation in the assay with 5 μM of C16CF3-coumarin. The final concentrations of compounds were 50 μM with 0.5% v/v DMSO in assay solution (20 μl). The enzymatic reaction fluorescence was monitored, and the slope was analyzed from the initial linear range (60 min) of reaction curves.

The hit compounds (10 mM in DMSO) were prepared at a stock concentration and diluted to 3.3, 1.1, 0.3, and 0.1 mM with DMSO. 200 nl of hit compounds with the mentioned concentrations were incubated with PLCγ2 (10 μl) for 15 min at room temperature. DMSO was used as the control. The assay reaction was commenced by adding 10 μl of C16CF3-coumarin. The final assay mixture contains compounds (33.75, 11.25, 3.75, and 1 μM), PLCγ2 (2.5 nM), and C16CF3-coumarin (5 μM). The assay reactions were monitored by detecting their fluorescence, and the slope was analyzed from the initial linear range (60 min) of reaction curves.

### Data analysis

Statistics were calculated using a One-way ANOVA with pairwise post hoc Tukey HSD comparison. Each experiment was repeated a minimum of three times. The data are shown as the mean ± standard deviation (SD).

## Results and discussion

### Synthesis of C16CF3-coumarin substrate

To create a PLCγ substrate that is amenable for a micellular assay, we endeavored to create a molecule that 1) would incorporate into a micelle, 2) could be enzymatically processed by PLCγ family enzymes to release a suitable signal for optical detection, and 3) would not be enzymatically processed in solution (i.e., no activity without the presence of micelles). To accomplish this, we first selected the known water-soluble substrate known as C8CF3-coumarin for chemical modification [22]. This substrate was chosen as it was an already optimized version of the original WH-15 PLC solution assay substrate that releases a "turn-on" fluorophore when processed by PLCγ enzymes.

**Fig 1:**
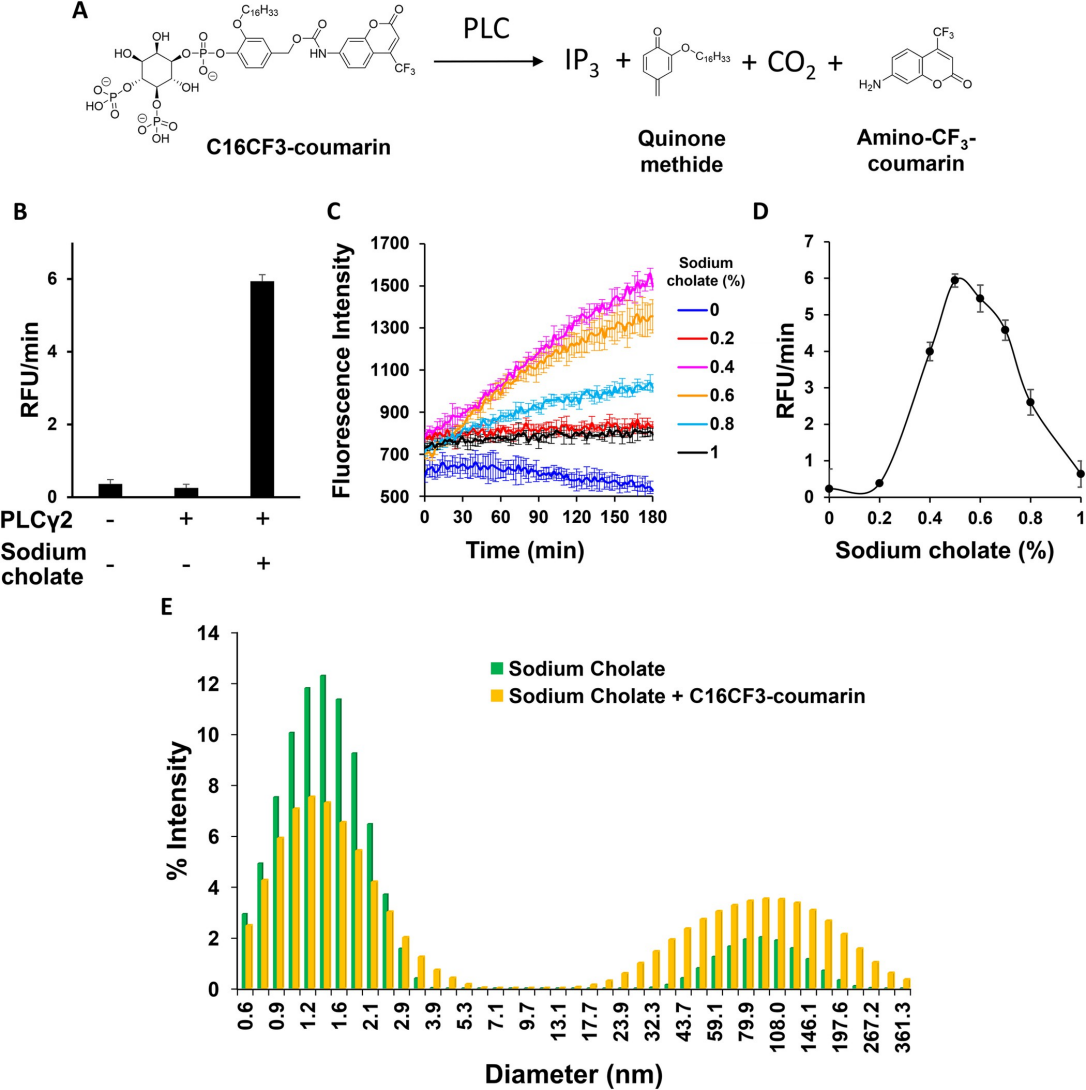
The significance of sodium cholate for the C16CF3-coumarin micelle assay. (A) Enzymatic activity upon the C16CF3-coumarin substrate. (B) Generation of fluorescence signal by C16CF3-coumarin (5 μM) in the presence or absence of sodium cholate (0.5% w/v) and/or PLCγ2 (2.5 nM) in solution assay buffer. (C) Enzyme reaction of C16CF3-coumarin (5 μM) by PLCγ2 (2.5 nM) in the presence of 0–1% (w/v) sodium cholate. (D) The slope (60 min) from the initial linear range of reaction profiles with 0–1% (w/v) sodium cholate. The values are shown as the mean ± SD from three different experiments. (E) The particle size distrubtion using Dynamic light scattering of sodium cholate (green) and sodium cholate plus C16CF3-coumarin micelles (yellow).

First, the C8CF3-coumarin substrate needed to become more hydrophobic to gain the necessary properties to be able to effectively incorporate into a micelle. Therefore, the C8-alkyl chain present in the original substrate was replaced with a longer C16-alkyl chain (**Scheme 1**). The "turn-on" 7-amino-4-trifluoromethyl-coumarin fluorophore then was installed to create the final substrate named C16CF3-coumarin (**Scheme 2**) utilizing similar chemistry to previous reports [22]. As with the original C8CF3-coumarin, the newly synthesized C16CF3-coumarin was expected to undergo the same cascade reaction upon enzymatic cleavage that releases the free fluorophore, as depicted in **Fig 1A**.

### Optimization of micelle assay conditions

Initially, the new C16CF3-coumarin substrate was tested in a solution assay where PLCγ2 and the substrate were directly added to the assay buffer, and the appearance of fluorescence signal from the liberated 7-amino-4-trifluoromethyl-coumarin was detected. As expected, no significant increase in fluorescence was detected (**Fig 1B**), thus confirming that this substrate cannot be efficiently processed in solution. The inability of the enzyme to process C16CF3-coumarin in the solution assay is likely due to the very small amount of free substrate actually present in solution due to the substrate’s hydrophobicity.

Next, to create a micellular assay using the C16CF3-coumarin substrate, an appropriate micelle-forming solubilizer needed to be identified. Bile salts appeared to be an ideal solubilizer as their natural function is to enhance the aqueous solubility of slightly soluble organic molecules through the presence of micelles [27]. Of the various bile salts, sodium cholate was chosen due to its common usage, commercial availability, and its ability to solubilize molecules with long alkyl chains [28].

With sodium cholate chosen as the micelle-forming solubilizer, the most optimal concentration that could form solubilizing micelles without detrimentally effecting the PLCγ enzyme’s processing was determined. As shown in **Fig 1C and 1D**, PLCγ2’s ability to process C16CF3-coumarin in the presence of 0 to 1% (w/v) sodium cholate was highly dependent upon sodium cholate concentration. As before, no enzymatic processing was observed at 0% (w/v) sodium cholate. An optimal concentration of 0.5% (w/v) was identified as increasing levels led to decreasing enzymatic processing of the substrate. This is most likely due to the increasing concentrations of sodium cholate impacting the structure of PLCγ2, resulting in an enzyme that is not catalytically proficient.

Next, the buffer conditions were further optimized for enzyme activity. As PLCγ enzymes are calcium-dependent, various concentrations of calcium chloride were tested, with an optimal concentration of 3 mM identified (**S1 Fig in S1 File**). Previously, BSA was shown to influence the reaction rate of the C8CF3-coumarin solution substrate [22], so various BSA concentrations were also tested to determine if they had any effect on the enzymatic processing of the C16CF3-coumarin micelle substrate. As shown in **S2 Fig in S1 File**, BSA had a modest effect on the ability of PLCγ2 to process the C16CF3-coumarin substrate, and a concentration of 0.04 mg/ml BSA was chosen. Thus, the final optimized assay conditions were as follows: C16CF3-coumarin (5 μM), 0.5% (w/v) sodium cholate, PLCγ2 (2.5 nM), BSA (0.04 mg/ml), and calcium chloride (3 mM).

### Confirmation of micelle formation

To ensure that the formation of micelles occurred under these conditions, dynamic light scattering was used to measure the presence and diameter of any micelles present in the presence or absence of the C16CF3-coumarin substrate. As shown in **Fig 1E**, sodium cholate alone formed single micelles with a diameter of ~1.5 nm, in good agreement with previous reports of sodium cholate micelles in similar buffer systems [29]. Sodium cholate micelles are also known to undergo secondary aggregation to form larger micellular aggregates, which can influence the incorporation of other molecules into the micelle [30]. Not surprisingly, these higher-order micellular aggregates are present as well (**Fig 1E**). When mixed micelles are formed with sodium cholate and C16CF3-coumarin, micelles of roughly the same diameters are formed, but the proportion of micelles in higher-order aggregates is increased slightly (**Fig 1E**). Taken together, the dynamic light scattering and enzymatic processing data confirm that micelles are indeed being formed, these sodium cholate micelles are essential to PLCγ2’s ability to process the C16CF3-coumarin substrate, and that the formed micelles provide a suitable environment for the enzymatic reaction.

### PLCγ enzyme family member reactivities and kinetics with C16CF3-coumarin

**Fig 2:**
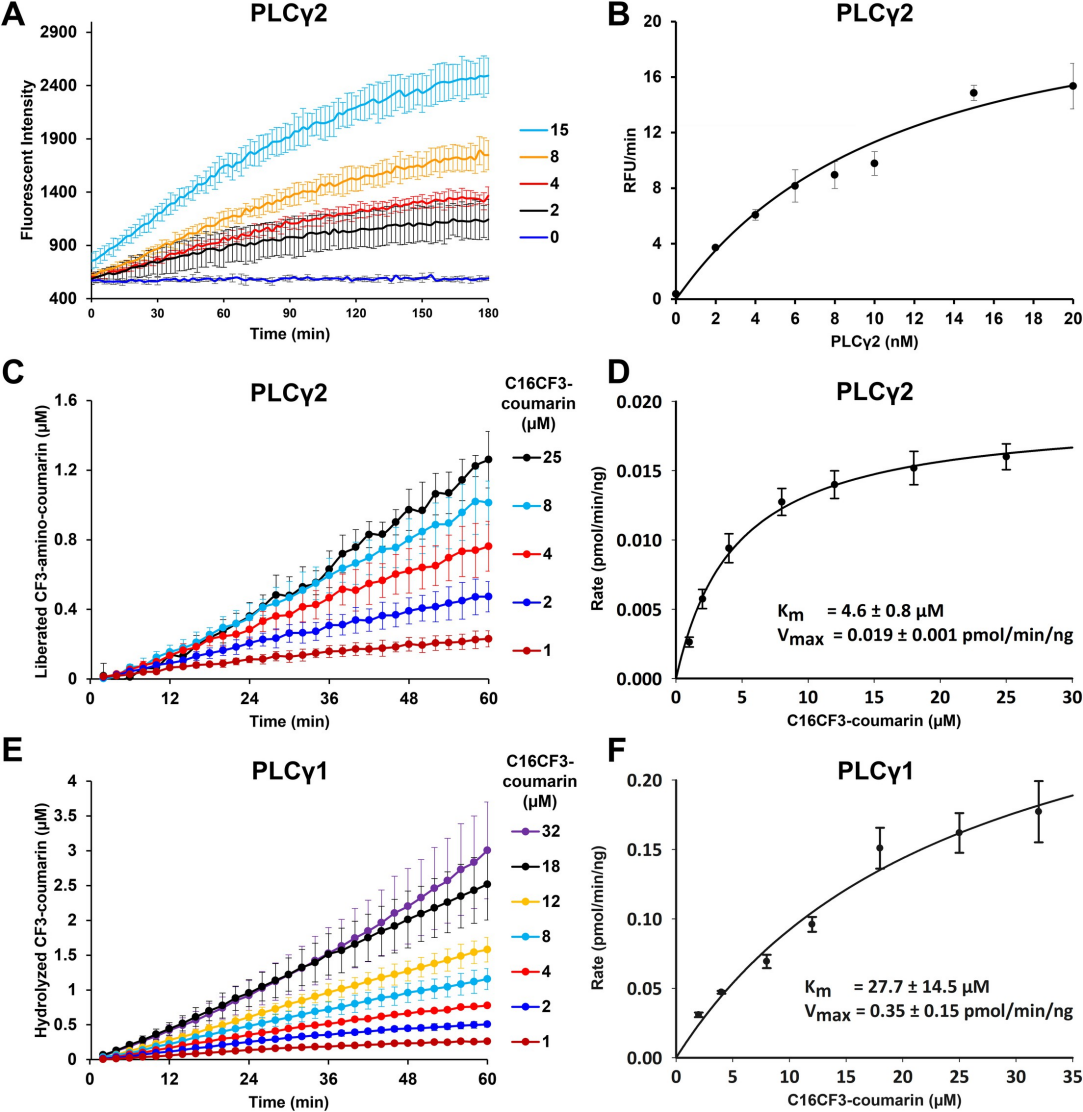
The enzymatic reaction of C16CF3-coumarin and PLCγ. (A) Enzymatic reaction profiles of C16CF3-coumarin (5 μM) with various concentrations of PLCγ2 (0–20 nM). (B) The initial linear range (60 min) of the reaction profiles was calculated for the slope. The values are shown as the mean ± SD from three different experiments. (C) PLCγ2 (10 nM) and varying concentrations of C16CF3-coumarin (1–25 μM) were added to the wells of a 384-well plate and fluorescence was monitored every two minutes. The initial linear range (60 min) of the reaction profiles was calculated for the slope. (D) Michalis-Menton Plot for C16CF3-coumarin kinetics with PLCγ2. The values are shown as the mean ± SD from three different experiments. (E) PLCγ1 (2 nM) and varying concentrations of C16CF3-coumarin (1–32 μM) were added to the wells of a 384-well plate and fluorescence was monitored every 2 minutes. The initial linear range (60 min) of the reaction profiles was calculated for the slope. (F) Michalis-Menton Plot for C16CF3-coumarin kinetics with PLCγ1. The values are shown as the mean ± SD from four different experiments.

With the optimal assay conditions determined, next, the enzyme reactivity of PLCγ2 with the C16CF3-coumarin substrate was tested in greater detail. As shown in **Fig 2A and 2B**, a dose-dependent response was observed with increasing enzyme concentrations over time, as expected. When the substrate concentration was varied, the reaction curves with PLCγ1 and PLCγ2 are shown in **Fig 2C–2F**. Since the exact configuration of the C16CF3-coumarin substrate in the micelles is not known, only apparent kinetic parameters can be calculated. These data yielded an apparent K_m_ of 27.7 ± 14.5 and 4.6 ± 0.8 μM and a V_max_ of 0.35 ± 0.15 and 0.019 ± 0.001 pmol/min/ng for PLCγ1 and PLCγ2, respectively (**Table 1**).

**Table 1:** Kinetic studies of C16CF3-coumarin with PLCγ enzyes.

	K_m_ (μM)	K_cat_ (min^-1^)	V_max_ (pmol/min/ng)	K_cat_ / K_m_ (μM^-1^•min^-1^)
PLCγ2				
C16CF3-coumarin	4.6 ± 0.8	2.7 ± 0.2	0.019 ± 0.001	0.6 ± 0.1
C8CF3-coumarin [22]	28.6 ± 5.6	175.6 ±62.2	1.24 ± 0.4	6.0 ± 1.1
PLCγ1				
C16CF3-coumarin	27.7 ± 14.5	50.0 ± 22.0	0.35 ± 0.15	1.96 ± 0.4
C8CF3-coumarin [22]	47.1 ± 13.5	214.9 ± 13.0	1.50 ± 0.1	4.8 ± 1.4
PIP2 [21]	28 ± 2.6	-	2.7 ± 0.1	-
WH-15	49 ± 7.2	-	4.2 ±0.3	-

As detailed in **Table 1**, the calculated apparent kinetic parameters reveal that the K_m_, K_cat_, V_max_, and K_cat_/K_m_ are all substantially lower for both PLCγ1 and PLCγ2 for the C16CF3-coumarin micellular substrate as compared to the solution C8CF3-coumarin substrate. Interestingly, the apparent K_m_ of the C16CF3-coumarin substrate for PLCγ1 is in very close agreement with its natural substrate, PIP_2_ [21,22]. Overall, the PLCγ enzymes appear to preferentially bind to the micellular version of the substrate, but the micelle substrate is processed far more slowly than the solution substrate.

### PLCγ enzyme activating mutations and inhibitors with C16CF3-coumarin

**Fig 3:**
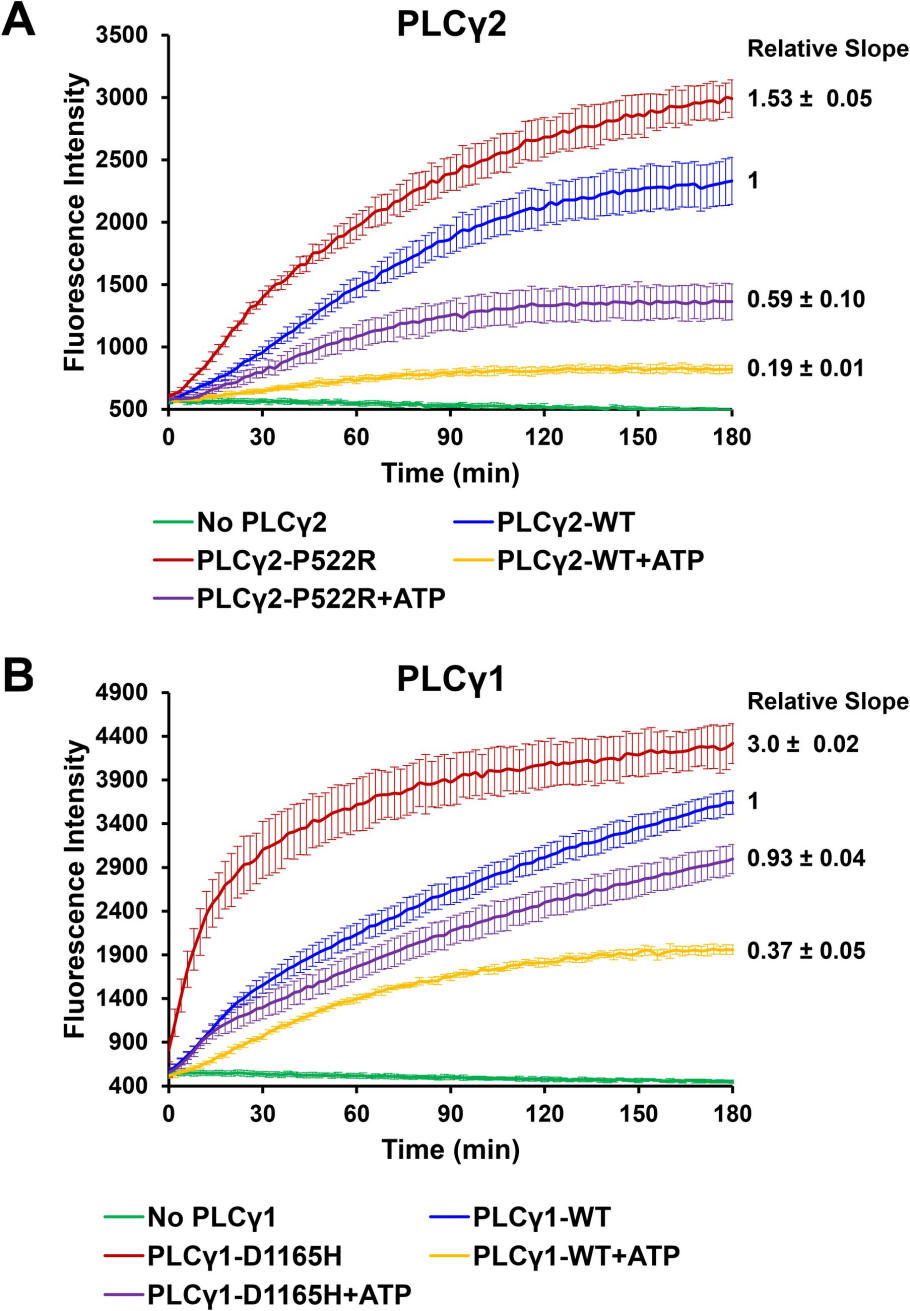
PLCγ enzyme activating mutation and inhibitor. (A) Enzymatic profile of no PLCγ2, PLCγ2-WT (2.5 nM) with and without ATP (4.8 mM), PLCγ2-P522R (2.5 nM) with and without ATP (4.8 mM), and C16CF3-coumarin (5 μM). (B) Enzymatic profile of no PLCγ1, PLCγ1-WT (2.5 nM) with and without ATP (4.8 mM), PLCγ2-D1165H (2.5 nM) with and without ATP (4.8 mM), and C16CF3-coumarin (5 μM). The values are shown as the mean ± SD from three different experiments.

Activating mutations for both PLCγ1 and PLCγ2 are known, and ATP has been shown to be an inhibitor of both enzymes [24]. To further validate the C16CF3-coumarin micelle assay, we measured the reaction of the substrate with the PLCγ1-D1665H and PLCγ2-P522R activating mutants in the presence or absence of ATP. As shown in **Fig 3A and 3B**, both PLCγ1-D1665H and PLCγ2-P522R were able to process the C16CF3-coumarin substrate faster than the corresponding wild-type enzymes, and ATP (4.8 mM) was able to inhibit these reactions as expected. The fold increase in activity of PLCγ1-D1665H to process the C16CF3-coumarin substrate over the wild-type PLCγ1 was similar to that of the previously reported XY-69 when used in a micelle assay; however, it was far less than the ~30-fold increase when XY-69 was formulated as a liposome [31]. Similarly, the fold increase in activity of the PLCγ2-P522R mutant over the wild-type enzyme for the C16CF3-coumarin substrate was similar to that of the previously reported increase in processing the C8CF3-courmain substrate in a solution assay [22]. These results indicate that the micelle assay could identify inhibitors and activators, but the liposomal XY-69 assay is likely still superior for identifying potential activators from a screen.

### High throughput screening of small molecules for PLCγ2 using C16CF3-coumarin

**Fig 4:**
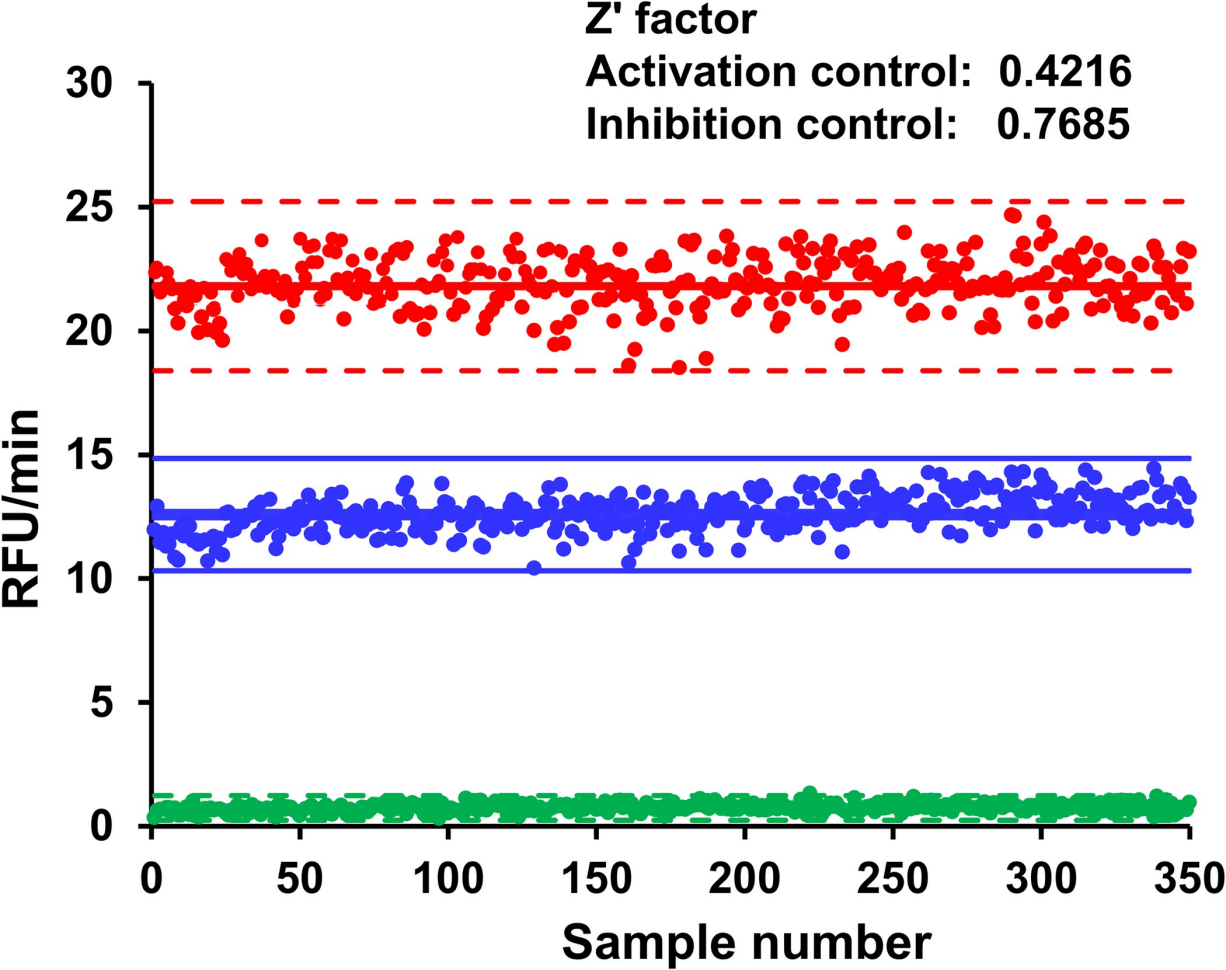
Z’ factor determination of PLCγ2 and C16CF3-coumarin. PLCγ2 at a concentration of 5.0 nM (red color; activation control), 2.5 nM (blue color; standard control), or 0 nM (green color; inhibition control) along with C16CF3-coumarin (5 μM) were added to all wells of a 384-well plate. Fluorescence intensity was measured every two minutes and the initial linear range (60 min) of the reaction profiles was calculated for the slope.

Before a small pilot screen of the LOPAC_1280_ compound library was performed, first, the Z’-factor was determined for the micelle assay. Wells with no PLCγ2 or a two-fold higher concentration of PLCγ2 (5 nM) were used as the inhibition and activation controls, respectively. As shown in **Fig 4**, Z’-factors of 0.422 were calculated for activation and 0.768 for inhibition. These values indicate that the optimized micelle assay conditions are amenable for the screening of activators, and the assay is in the “excellent assay” range for the screening of inhibitors.

Next, a pilot screen of 1280 compounds from the Library of Pharmacologically Active Compounds (50 μM) was carried out to identify potential compounds that could significantly increase or decrease the enzymatic activity of PLCγ2. A relatively high concentration of test compounds was used to explore how common assay interference from the compounds would be with this assay type. After the screening, the top ten inhibitor and activator compounds were tested in a 5-point dose-response assay (1–100 μM) using the same C16CF3-coumarin substrate.

**Fig 5:**
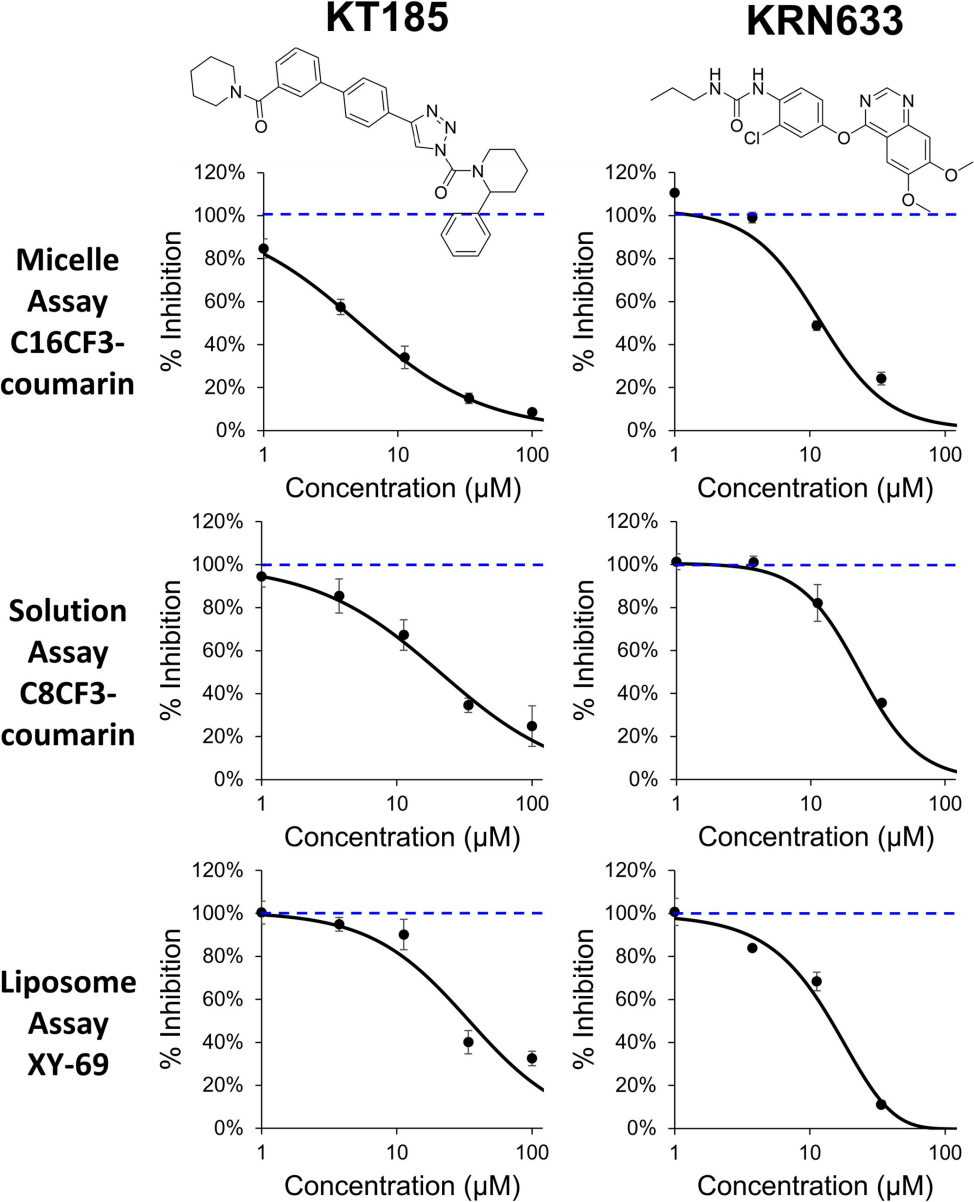
LOPAC_1280_ library screening. Dose-response profiles of selected hit inhibitor compounds from the primary screening of the LOPAC_1280_ library. Various concentrations of the hit compounds (1 to 100 μM) were added to the wells of a 384-well plate along with 2.5 nM PLCγ2 and 5 μM C16CF3-coumarin. Fluorescence intensity was monitored every two minutes and the initial linear range (60 min) of the reaction profiles was calculated for the slope. The % inhibition was calculated using the values from the slope of PLCγ2 alone (0% inhibition) and wells containing no PLCγ2 (100% inhibition). The values are shown as the mean SD from three different experiments. Each experiment had triplicate wells.

All ten inhibitors reproduced and exhibited a reasonable dose-response curve except Aurintricarboxylic acid, a known promiscuous binder [24,32] that completely inhibited all enzymatic activity at all concentrations tested. **Fig 5** shows two representative inhibitors: KT185 and KRN633. These two inhibitors were then tested using the C8CF3-coumarin substrate in a solution assay and the XY-69 substrate in a liposome assay. As shown in **Fig 5**, the three assay types were in relatively good agreement for these inhibitors.

For the activators, only five of the top 10 hits reproduced and showed a dose-response profile (**S3 Fig in S1 File**). These compounds were S-(+)-Fluoxetine hydrochloride, Amitriptyline hydrochloride, Octoclothepin, Triflupromazine hydrochloride, and Tamoxifen. When these compounds were tested using the C8CF3-coumarin substrate in the solution assay, none exhibited any activation. While this result could suggest that an activation mechanism of action of these compounds requires a micelle or liposome, unfortunately, none of these compounds reproduced their activation activity in the XY-69 liposomal assay either (**S4 Fig in S1 File**). Currently, it is unclear why there are such disparate results between the C16CF3-coumarin micelle and XY-69 liposomal assays; however, these data suggest that the XY-69 liposomal assay may be the preferred method for screening potential PLCγ enzyme family activators. It is intriguing to speculate on the mechanism of action of the activators, given that all five dose-response active hits have a cationic amphiphilic structure, and four are reported inducers of phospholipidosis [33–35]. Such compounds are known to disrupt membranes through their effects on phospholipid processing and structure. These toxicity-inducing effects are non-target specific and therefore it has been suggested to avoid progressing such structures [36]. As for the differences in the compounds’ activities between micelle and liposomal assays, it is clear that the unique chemical makeup of the micelles and liposomes certainly elicits different interactions to produce varied effects on enzymatic activity.

## Conclusions

Aberrant activity of PLC proteins, specifically the PLCγ enzyme family, has been implicated in many diseases. Therefore, the identification of selective modulators of these enzymes to identify chemical probes to study disease processes and to develop potential therapeutics is still needed. In this study, a new PLC substrate, C16CF3-coumarin, was developed, and a micellular-based assay was optimized to identify small molecule modulators of PLC enzymes. A pilot screen of the LOPAC_1280_ compound library was performed using this micellular assay. Identified hits that reproduced and exhibited a dose-dependence in the micelle assays were tested in a solution assay using the previously developed C8CF3-coumarin substrate [22] and in a liposomal assay using XY-69 [23]. The micelle assay showed good agreement with the solution and liposomal assays for identified inhibitors. However, activators did not reproduce across the two assay types. Our work reported here indicates that the micelle assay using the new C16CF3-coumarin substrate has advantages over other assay types reported to date for the identification of PLC enzyme inhibitors.

## Supporting information

S1 File The SI contains detailed synthetic procedures with corresponding NMR spectra, optimization curves for calcium and BSA, along with dose-response profiles of selected activator compounds in the micellular and liposomal assay. (PDF)

## References

[1] Bill CA, Vines CM. Phospholipase C. 2020. pp. 215–242. doi: 10.1007/978-3-030-12457-1_9 31646512 PMC7790445

[2] Kadamur G, Ross EM. Mammalian Phospholipase C. Annu Rev Physiol. 2013;75: 127–154. doi: 10.1146/annurev-physiol-030212-183750 23140367

[3] Magno L, Lessard CB, Martins M, Lang V, Cruz P, Asi Y, et al. Alzheimer’s disease phospholipase C-gamma-2 (PLCG2) protective variant is a functional hypermorph. Alzheimers Res Ther. 2019;11: 16. doi: 10.1186/s13195-019-0469-0 30711010 PMC6359863

[4] Jackson JT, Mulazzani E, Nutt SL, Masters SL. The role of PLCγ2 in immunological disorders, cancer, and neurodegeneration. J Biol Chem. 2021;297: 100905. doi: 10.1016/j.jbc.2021.100905 34157287 PMC8318911

[5] Koss H, Bunney TD, Behjati S, Katan M. Dysfunction of phospholipase Cγ in immune disorders and cancer. Trends Biochem Sci. 2014;39: 603–611. doi: 10.1016/j.tibs.2014.09.004 25456276

[6] Tao P, Han X, Wang Q, Wang S, Zhang J, Liu L, et al. A gain-of-function variation in PLCG1 causes a new immune dysregulation disease. J Allergy Clin Immunol. 2023. doi: 10.1016/j.jaci.2023.06.020 37422272 PMC10770301

[7] Abe K, Fuchs H, Boersma A, Hans W, Yu P, Kalaydjiev S, et al. A novel N-ethyl-N-nitrosourea-induced mutation in phospholipase Cγ2 causes inflammatory arthritis, metabolic defects, and male infertility in vitro in a murine model. Arthritis Rheum. 2011;63: 1301–1311. doi: 10.1002/art.30280 21305534

[8] Vaqué JP, Gómez-López G, Monsálvez V, Varela I, Martínez N, Pérez C, et al. PLCG1 mutations in cutaneous T-cell lymphomas. Blood. 2014;123: 2034–2043. doi: 10.1182/blood-2013-05-504308 24497536

[9] Prawiro C, Bunney TD, Kampyli C, Yaguchi H, Katan M, Bangham CRM. A frequent PLCγ1 mutation in adult T-cell leukemia/lymphoma determines functional properties of the malignant cells. Biochim Biophys Acta—Mol Basis Dis. 2023;1869: 166601. doi: 10.1016/j.bbadis.2022.166601 36442790

[10] Walliser C, Hermkes E, Schade A, Wiese S, Deinzer J, Zapatka M, et al. The Phospholipase Cγ2 Mutants R665W and L845F Identified in Ibrutinib-resistant Chronic Lymphocytic Leukemia Patients Are Hypersensitive to the Rho GTPase Rac2 Protein. J Biol Chem. 2016;291: 22136–22148. doi: 10.1074/jbc.M116.746842 27542411 PMC5063995

[11] Liu T-M, Woyach JA, Zhong Y, Lozanski A, Lozanski G, Dong S, et al. Hypermorphic mutation of phospholipase C, γ2 acquired in ibrutinib-resistant CLL confers BTK independency upon B-cell receptor activation. Blood. 2015;126: 61–68. doi: 10.1182/blood-2015-02-626846 25972157 PMC4492196

[12] Huynh MQ, Goßmann J, Gattenlöehner S, Klapper W, Wacker H-H, Ramaswamy A, et al. Expression and pro-survival function of phospholipase Cγ2 in diffuse large B-cell lymphoma. Leuk Lymphoma. 2015;56: 1088–1095. doi: 10.3109/10428194.2014.941832 25012946

[13] Li T, Yang Z, Li H, Zhu J, Wang Y, Tang Q, et al. Phospholipase Cγ1 (PLCG1) overexpression is associated with tumor growth and poor survival in IDH wild-type lower-grade gliomas in adult patients. Lab Investig. 2022;102: 143–153. doi: 10.1038/s41374-021-00682-7 34697421 PMC8784314

[14] Gossmann J, Stolte M, Lohoff M, Yu P, Moll R, Finkernagel F, et al. A Gain-Of-Function Mutation in the Plcg2 Gene Protects Mice from Helicobacter felis-Induced Gastric MALT Lymphoma. Gangopadhyay N, editor. PLoS One. 2016;11: e0150411. doi: 10.1371/journal.pone.0150411 26966907 PMC4788355

[15] Sims R, van der Lee SJ, Naj AC, Bellenguez C, Badarinarayan N, Jakobsdottir J, et al. Rare coding variants in PLCG2, ABI3, and TREM2 implicate microglial-mediated innate immunity in Alzheimer’s disease. Nat Genet. 2017;49: 1373–1384. doi: 10.1038/ng.3916 28714976 PMC5669039

[16] Conway OJ, Carrasquillo MM, Wang X, Bredenberg JM, Reddy JS, Strickland SL, et al. ABI3 and PLCG2 missense variants as risk factors for neurodegenerative diseases in Caucasians and African Americans. Mol Neurodegener. 2018;13: 53. doi: 10.1186/s13024-018-0289-x 30326945 PMC6190665

[17] van der Lee SJ, Conway OJ, Jansen I, Carrasquillo MM, Kleineidam L, van den Akker E, et al. A nonsynonymous mutation in PLCG2 reduces the risk of Alzheimer’s disease, dementia with Lewy bodies and frontotemporal dementia, and increases the likelihood of longevity. Acta Neuropathol. 2019;138: 237–250. doi: 10.1007/s00401-019-02026-8 31131421 PMC6660501

[18] Dalmasso MC, Brusco LI, Olivar N, Muchnik C, Hanses C, Milz E, et al. Transethnic meta-analysis of rare coding variants in PLCG2, ABI3, and TREM2 supports their general contribution to Alzheimer’s disease. Transl Psychiatry. 2019;9: 55. doi: 10.1038/s41398-019-0394-9 30705288 PMC6355764

[19] Rusten TE, Stenmark H. Analyzing phosphoinositides and their interacting proteins. Nat Methods. 2006;3: 251–258. doi: 10.1038/nmeth867 16554828

[20] Perrella FW, Chen S-F, Behrens DL, Kaltenbach RFI, Seitz SP. Phospholipase C Inhibitors: A New Class of Agents. J Med Chem. 1994;37: 2232–2237. doi: 10.1021/jm00040a016 8035430

[21] Huang W, Hicks SN, Sondek J, Zhang Q. A Fluorogenic, Small Molecule Reporter for Mammalian Phospholipase C Isozymes. ACS Chem Biol. 2011;6: 223–228. doi: 10.1021/cb100308n 21158426 PMC3312000

[22] Visvanathan R, Utsuki T, Beck DE, Lendy E, Sun K, Liu Y, et al. A novel fluorogenic reporter substrate for 1-phosphatidylinositol 4,5-bisphosphate phosphodiesterase gamma-2 (PLCγ2): Application to high-throughput screening for activators to treat Alzheimer’s disease. SLAS Discov. 2023;28: 170–179. doi: 10.1016/j.slasd.2023.03.003 36933698 PMC10251139

[23] Carr AJ, Siraliev-Perez E, Huang W, Sondek J, Zhang Q. Fluorogenic XY-69 in Lipid Vesicles for Measuring Activity of Phospholipase C Isozymes. 2021. pp. 225–236. doi: 10.1007/978-1-0716-1142-5_17 33481244 PMC8094607

[24] Huang W, Carr AJ, Hajicek N, Sokolovski M, Siraliev-Perez E, Hardy PB, et al. A High-Throughput Assay to Identify Allosteric Inhibitors of the PLC-γ Isozymes Operating at Membranes. Biochemistry. 2020;59: 4029–4038. doi: 10.1021/acs.biochem.0c00511 33028071 PMC8081235

[25] Huang W, Barrett M, Hajicek N, Hicks S, Harden TK, Sondek J, et al. Small Molecule Inhibitors of Phospholipase C from a Novel High-throughput Screen*. J Biol Chem. 2013;288: 5840–5848. doi: 10.1074/jbc.M112.422501 23297405 PMC3581404

[26] Bjørsvik H-R, Liguori L, Minisci F. High Selectivity in the Oxidation of Mandelic Acid Derivatives and in O- Methylation of Protocatechualdehyde: New Processes for Synthesis of Vanillin, i so- Vanillin, and Heliotropin. Org Process Res Dev. 2000;4: 534–543. doi: 10.1021/op0000529

[27] Malik NA. Solubilization and Interaction Studies of Bile Salts with Surfactants and Drugs: a Review. Appl Biochem Biotechnol. 2016;179: 179–201. doi: 10.1007/s12010-016-1987-x 26781714

[28] Sugioka H, Moroi Y. Micelle formation of sodium cholate and solubilization into the micelle. Biochim Biophys Acta—Lipids Lipid Metab. 1998;1394: 99–110. doi: 10.1016/s0005-2760(98)00090-3 9767136

[29] Maslova VA, Kiselev MA. Structure of Sodium Cholate Micelles. Crystallogr Reports. 2018;63: 472–475. doi: 10.1134/S1063774518030173

[30] Li G, McGown LB. Model for Bile Salt Micellization and Solubilization from Studies of a “Polydisperse” Array of Fluorescent Probes and Molecular Modeling. J Phys Chem. 1994;98: 13711–13719. doi: 10.1021/j100102a043

[31] Hajicek N, Keith NC, Siraliev-Perez E, Temple BR, Huang W, Zhang Q, et al. Structural basis for the activation of PLC-γ isozymes by phosphorylation and cancer-associated mutations. Elife. 2019;8. doi: 10.7554/eLife.51700 31889510 PMC7004563

[32] Nguyen TG, Honson NS, Arns S, Davis TL, Dhe-Paganon S, Kovacic S, et al. Development of Fluorescent Substrates and Assays for the Key Autophagy-Related Cysteine Protease Enzyme, ATG4B. Assay Drug Dev Technol. 2014;12: 176–189. doi: 10.1089/adt.2013.561 24735444 PMC3994995

[33] Muehlbacher M, Tripal P, Roas F, Kornhuber J. Identification of Drugs Inducing Phospholipidosis by Novel in vitro Data. ChemMedChem. 2012;7: 1925–1934. doi: 10.1002/cmdc.201200306 22945602 PMC3533795

[34] Shahane SA, Huang R, Gerhold D, Baxa U, Austin CP, Xia M. Detection of Phospholipidosis Induction: A Cell-Based Assay in High-Throughput and High-Content Format. SLAS Discov. 2014;19: 66–76. doi: 10.1177/1087057113502851 24003057 PMC4550094

[35] Zhang X, Yang L, Liu Y, Song Z, Zhao J, Chen D, et al. Detection of nanocarrier potentiation on drug induced phospholipidosis in cultured cells and primary hepatocyte spheroids by high content imaging and analysis. Toxicol Appl Pharmacol. 2018;348: 54–66. doi: 10.1016/j.taap.2018.04.016 29678448 PMC6716368

[36] Tummino TA, Rezelj V V., Fischer B, Fischer A, O’Meara MJ, Monel B, et al. Drug-induced phospholipidosis confounds drug repurposing for SARS-CoV-2. Science (80-). 2021;373: 541–547. doi: 10.1126/science.abi4708 34326236 PMC8501941

