# Supplementary material for: A novel micellular fluorogenic substrate for quantitating the activity of 1-phosphatidylinositol 4,5-bisphosphate phosphodiesterase gamma (PLCγ) enzymes
Source: PLoS One. 2024 Mar 29;19(3):e0299541. doi: 10.1371/journal.pone.0299541 (PMC10980208; doi:10.1371/journal.pone.0299541)

A Novel Micellular Fluorogenic Substrate for Quantitating the Activity of 1-Phosphatidylinositol 4,5-bisphosphate Phosphodiesterase Gamma (PLCγ) Enzymes

Ramya Visvanathan[1,2,3], Tadanobu Utsuki[1,2], Daniel E. Beck[1,2], W. Brent Clayton[2,4], Emma Lendy[2,5], Kuai-lin Sun[6], Yinghui Liu[6], Kirk W. Hering[6], Andrew Mesecar[2,5], Zhong-Yin Zhang[1,2,3], Karson S. Putt[1,2,*]

[1] Institute for Drug Discovery, Purdue University, West Lafayette IN 47907 USA

[2] IUSM-Purdue TREAT-AD Center, West Lafayette IN 47907 USA

[3] Department of Medicinal Chemistry and Molecular Pharmacology, Purdue University, West Lafayette IN 47907 USA

[4] Division of Clinical Pharmacology, Indiana University School of Medicine, Indianapolis IN 46202 USA

[5] Department of Biochemistry, Purdue University, West Lafayette IN 47907 USA

[6] Cayman Chemical Company, 1180 East Ellsworth Road, Ann Arbor MI 48108 USA

*Author to whom all correspondence should be addressed:

Karson Putt

Email: puttk@purdue.edu

Phone: 765-494-7125

Institute for Drug Discovery

Purdue University

720 Clinic Drive

West Lafayette IN 47907

Table of Contents

Synthesis of C8CF16-coumarin **3**

Chemistry General Procedures 3

Intermediate 2 3

^{1}H NMR 4

Intermediate 3 5

^{1}H NMR 6

^{31}P NMR 7

Intermediate 5 8

^{1}H NMR 9

^{31}P NMR 10

Intermediate 6 11

^{1}H NMR 12

^{31}P NMR 14

Intermediate 9 16

^{1}H NMR 17

C16CF3-coumarin (Compound 10) 20

^{1}H NMR 21

^{19}F NMR 24

^{31}P NMR 25

SI Figure 1. Calcium optimizations for C16CF3-coumarin assay 26

SI Figure 2. BSA optimizations for C16CF3-coumarin assay 27

SI Figure 3. Dose-response profiles of selected activator compounds from the LOPAC1280 screen 28

SI Figure 4. Dose-response profiles of selected micelle activator compounds in the XY-69 assay 29

Chemistry General Procedures:

Reagents and solvents were purchased from commercial sources and used without further purification, except where indicated. Widely practiced methods were followed to conduct, analyze, and purify reactions, including techniques for exclusion of moisture and oxygen where appropriate. 4 Å molecular sieves were prepared for use by drying at 110 °C overnight, and were cooled to rt under N_2 atmosphere immediately prior to use. TMSBr was distilled (bp 79 °C) at atmospheric pressure under N_2 atmosphere. NMR experiments were performed on 400 MHz JEOL (400 MHz for ^{1}H, 162 MHz for ^{31}P) or 500 MHz Bruker (500 MHz for ^{1}H, 470 MHz for ^{19}F, 202 MHz for ^{31}P) instruments. NMR data were processed using MestReNova software. NMR samples were generally prepared at a concentration of 5 mg compound in 0.75 mL indicated deuterated solvent. For ^{1}H NMR, chemical shifts in ppm relative to the residual solvent peak, multiplicities, coupling constants in Hertz, and numbers of protons are indicated. For ^{19}F and ^{31}P NMR, chemical shifts in ppm relative to an external standard are indicated. Analytical TLC was performed on F254 silica gel plates, and 254 nm UV light, I_2 staining, and/or 10% H_2SO_4 staining were used for visualization. NP flash LC was performed on a Teledyne-ISCO NextGen300 instrument using prepacked silica gel columns available from Teledyne-ISCO, or in glass columns using loose SilicaFlash F60 silica gel. RP flash LC was performed on a Teledyne-ISCO NextGen300 instrument using prepacked C8-derivatized silica gel columns available from Teledyne-ISCO. Analytical LC-MS data were obtained on (1) a Waters ACQUITY UPLC equipped with Waters ACQUITY UPLC BEH C18 column (1.7 µm, 50 mm x 2.1 mm), TUV dual-wavelength detector, and SQD2 mass detector (ESI), or (2) an Agilent 1200 HPLC equipped with Agilent Zorbax SB-C8 column (5 µm, 50 mm x 2.1 mm) or Waters Atlantis T3 C18 column (3µm, 50 mm x 3.0 mm), 1100 Diode Array Detector G1315B, and 6130A Quadrupole mass detector (ESI and APCI). High-resolution mass spectra were obtained on Agilent 6550 Q-TOF or Thermo Q Exactive instruments.

Intermediate 2. A solution of 3,4-dihydroxybenzaldehyde (**1**, 3.00 g, 21.7 mmol, 1.4 eq) in DMF (10 mL) was added dropwise to a 0 ˚C solution of 1-bromohexadecane (4.64 g, 15.2 mmol, 1 eq) in DMF (20 mL) and the reaction was stirred for 30 min. Then, a solution of a 60% oil dispersion of NaH (2.15 g, 53.8 mmol, 3.5 eq) in THF (20 mL) was added dropwise and the reaction was allowed to slowly warm to rt while stirring for 48 h. The reaction was then warmed to 50 ˚C with stirring for 3 h upon which it was quenched with H_2O (50 mL) and extracted with EtOAc (3 x 50 mL). The combined organic phases were washed with brine (50 mL) and concentrated to give an oily residue. The residue was purified by flash LC on a silica gel column, eluting with 97:3 to 92:8 heptane:EtOAc gradient, to provide **2** (2.10 g, 38%) as a white solid. TLC R_f = 0.74 (80:20 heptane:EtOAc); ^{1}H NMR ($CDCl_3$, 400 MHz) δ 9.80 (s, 1H), 7.4 – 7.4 (m, 2H), 7.03 (d, *J* = 8.5 Hz, 1H), 6.22 (s, 1H), 4.10 (t, *J* = 6.6 Hz, 2H), 1.9 – 1.8 (m, 2H), 1.5 – 1.4 (m, 2H), 1.4 – 1.2 (m, 24H), 0.86 (t, *J* = 5.3 Hz, 3H); ESI-HRMS for $[M+H]^+$ $C_{23}H_{39}O_3$: calcd 363.2899, found 363.2889.

Compound 2

Compound 12 C16

Item #

Batch #

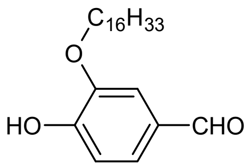

File Name	\\sulfur\private\nmrdata\JEOL_2022\KS2224-5\KS2224-5_PROTON_26-Apr-2022-1-1.jdf				
Date	26 Apr 2022 09:55:57	***Nucleus***	1H	***Frequency (MHz)***	399.5822
Solvent	CHLOROFORM-d	***Number of Transients***	16	***Origin***	JEOL ECZ400S Sc v601
Temperature (degree C)	20.200				

^{1}H NMR (CHLOROFORM-d, 400 MHz) δ 9.80 (s, 1H), 7.4-7.4 (m, 2H), 7.03 (d, 1H, *J*=8.5 Hz), 6.22 (s, 1H), 4.10 (t, 2H, *J*=6.6 Hz), 1.8-1.9 (m, 2H), 1.4-1.5 (m, 2H), 1.2-1.4 (m, 24H), 0.86 (t, 3H, *J*=5.3 Hz)

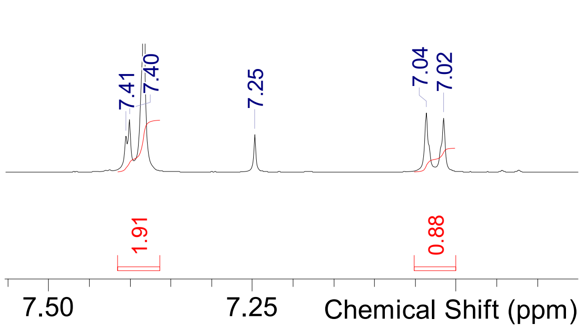

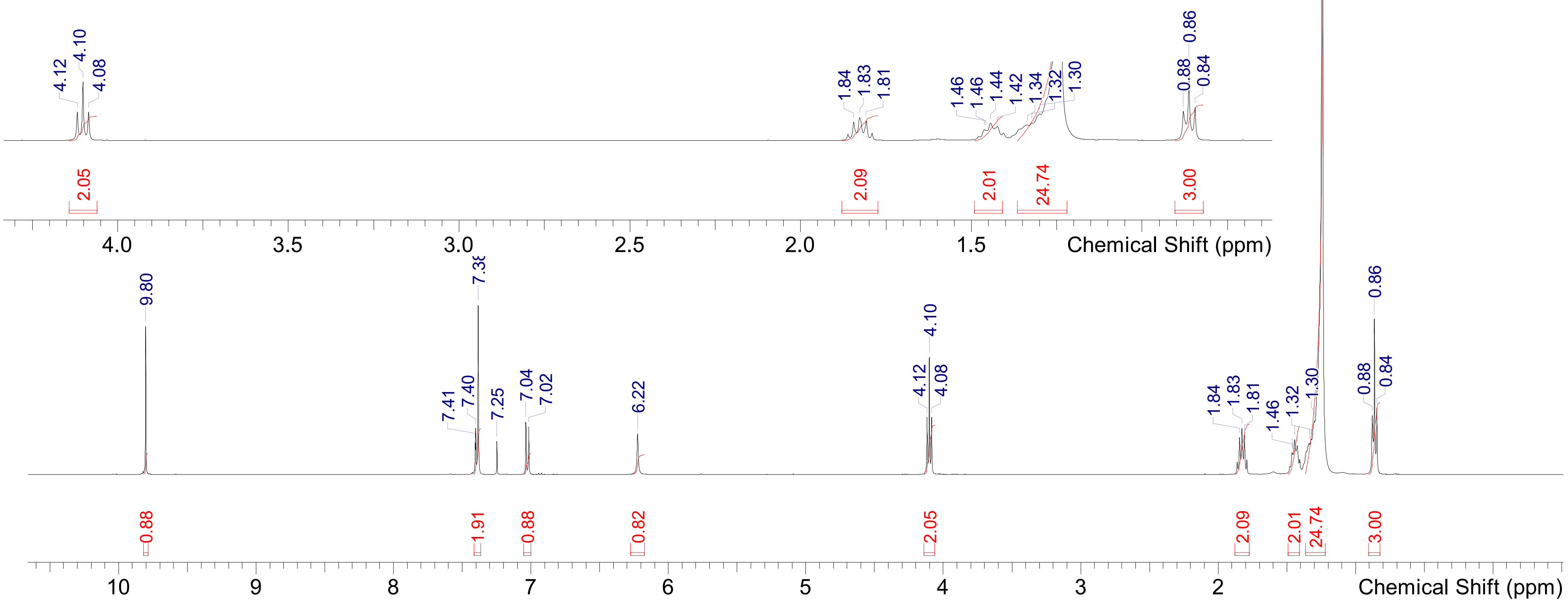

Intermediate 3. [*Note: as mentioned in the manuscript, it was advantageous to perform a one-pot procedure to access **5** from **2**, without isolation of **3**. This procedure and characterization of **3** was available from earlier synthetic investigations.*] 1*H*-Tetrazole (1.35 mL of a 0.45 M solution in ACN, 0.608 mmol, 1.1 eq) was added to a solution of **2** (200 mg, 0.552 mmol, 1 eq) in THF (20 mL), the reaction cooled to 0 ˚C, and a solution of benzyl N,N,N',N'-tetraisopropylphosphorodiamidite (200 mg, 0.591 mmol, 1.1 eq) in THF (2 mL) was added by syringe. The reaction was allowed to slowly warm to rt while stirring for 16 h. It was then concentrated to remove solvent and directly purified by flash LC on a silica gel column, eluting with 1% NEt_3 in heptane, to provide **3** (200 mg, 60%) as a colorless oil. TLC R_f = 0.72 (75:25 heptane:EtOAc); ^{1}H NMR ($CDCl_3$, 400 MHz) δ 9.84 (s, 1H), 7.4 – 7.2 (m, 7H), 7.18 (dd, *J* = 1.8, 8.0 Hz, 1H), 4.9 – 4.8 (m, 2H), 4.00 (t, *J* = 6.5 Hz, 2H), 3.9 – 3.7 (m, 2H), 1.8 – 1.7 (m, 2H), 1.5 – 1.4 (m, 2H), 1.3 – 1.2 (m, 36H), 0.87 (t, *J* = 6.8 Hz, 3H); ^{31}P NMR ($CDCl_3$, 162 MHz) δ 148.23; ESI-HRMS for $[M+H]^+$ $C_{36}H_{59}NO_4P$: calcd 600.4182, found 600.4173.

Compound 3

YL2035-178-1

Item #

Batch #

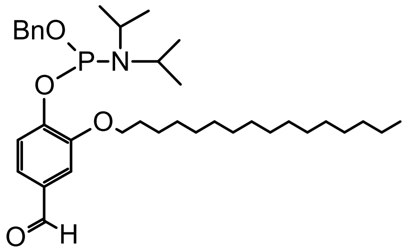

File Name	\\sulfur\private\nmrdata\JEOL_2022\YL2035-178-1\YL2035-178-1_PROTON_27-Apr-2022-1-1.jdf				
Date	27 Apr 2022 16:28:55	***Nucleus***	1H	***Frequency (MHz)***	399.7822
Solvent	CHLOROFORM-d	***Number of Transients***	16	***Origin***	JEOL ECZ400S Ca v601
Temperature (degree C)	21.000				

^{1}H NMR (CHLOROFORM-d, 400 MHz) δ 9.84 (s, 1H), 7.2-7.4 (m, 7H), 7.18 (dd, 1H, *J*=1.8, 8.0 Hz), 4.8-4.9 (m, 2H), 4.00 (t, 2H, *J*=6.5 Hz), 3.7-3.9 (m, 2H), 1.7-1.8 (m, 2H), 1.4-1.5 (m, 2H), 1.2-1.3 (m, 36H), 0.87 (t, 3H, *J*=6.8 Hz)

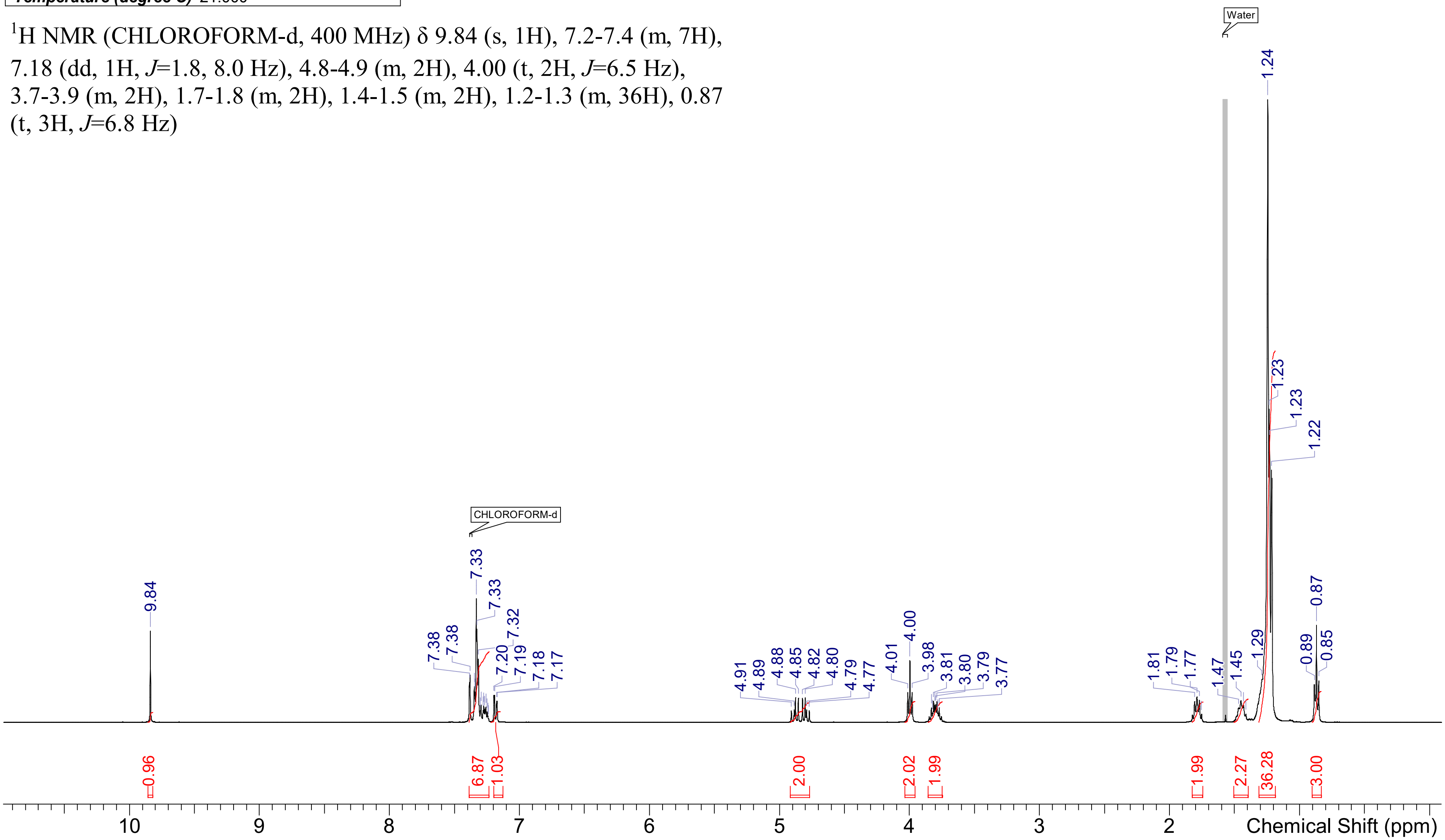

Compound 3

YL2035-178-1

Item #

Batch #

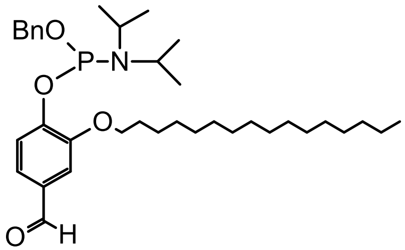

File Name	\\sulfur\private\nmrdata\JEOL_2022\YL2035-178-1\YL2035-178-1_PHOSPHORUS_27-Apr-2022-1-1.jdf				
Date	27 Apr 2022 16:45:05	***Nucleus***	31P	***Frequency (MHz)***	161.8347
Solvent	CHLOROFORM-d	***Number of Transients***	16	***Origin***	JEOL ECZ400S Ca v601
Temperature (degree C)	21.000				

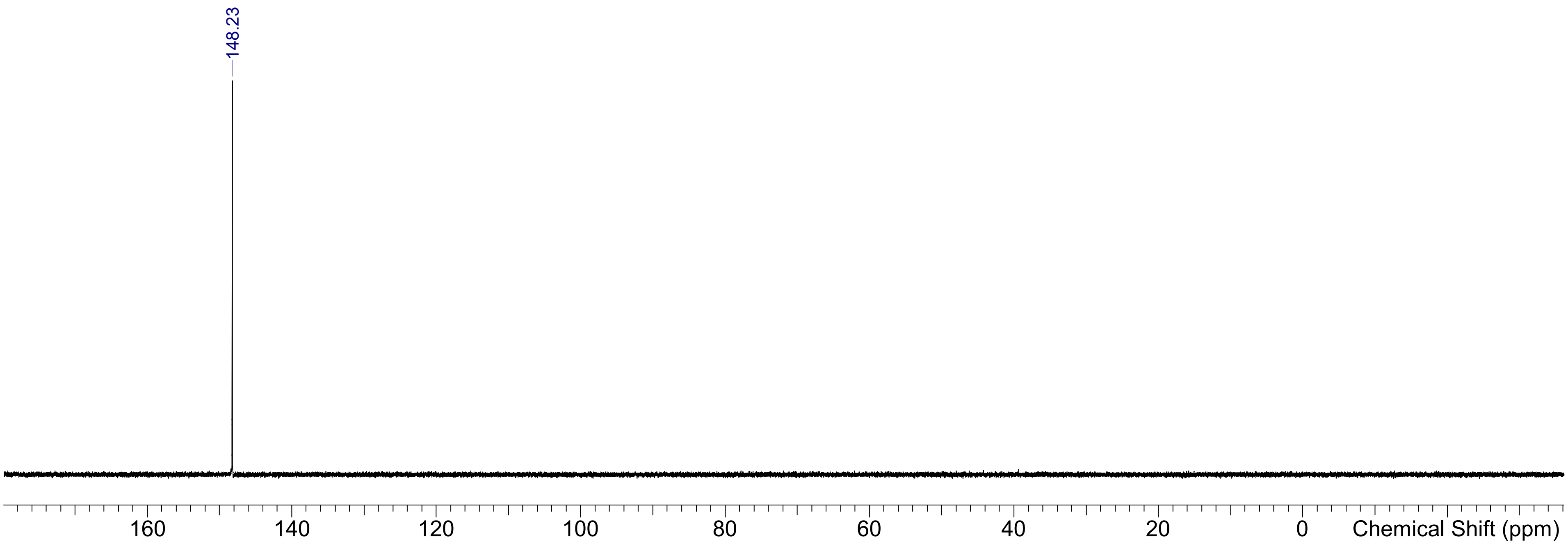

Intermediate 5. 1*H*-Tetrazole (22 mL of a 0.45 M solution in ACN, 9.9 mmol, 1.6 eq) was added to a solution of **2** (2.578 g, 7.111 mmol, 1.1 eq) in THF (60 mL), the reaction cooled to 0 ˚C and benzyl N,N,N',N'-tetraisopropylphosphorodiamidite (2.64 g, 7.80 mmol, 1.3 eq) was added by syringe. The reaction was stirred at 0 ˚C for 2 h, then was warmed to rt, giving unisolated **3**. A solution of alcohol **4** (5.20 g, 6.24 mmol, 1 eq) in THF (20 mL) was added followed by 1*H*-tetrazole (16 mL of a 0.45 M solution in ACN, 7.2 mmol, 1.2 eq) and the reaction was heated to 30 ˚C with stirring for 16 h. TLC indicated that the reaction was not yet complete so the temperature was increased to 35 ˚C and the reaction was allowed to stir for an additional 24 h. At this point, this phase of the reaction was deemed complete so it was cooled to 0 ˚C and *t*-BuOOH solution (1.20 mL of a 70 wt% solution in H_2O, 8.67 mmol, 1.4 eq) was added and stirred at 0 ˚C for 1 h. TLC showed partial reaction for this step so additional *t*-BuOOH solution (1.20 mL of a 70 wt% solution in H_2O, 8.67 mmol, 1.4 eq) was added and stirred at rt for 2 h. The reaction was then determined to be complete and was partitioned in H_2O (100 mL) and EtOAc (100 mL). The phases were separated and the organic phase was washed with 10% aqueous $Na_2S_2O_3$ solution (50 mL) and concentrated to give an oily residue. The residue was purified by flash LC on a silica gel column, eluting with 100:0 to 25:75 heptane:EtOAc gradient, to provide **5** (3.90 g, 46%) as a colorless oil. TLC R_f = 0.21 (40:60 heptane:EtOAc); ^{1}H NMR ($CDCl_3$, 400 MHz) δ 9.89 (d, *J* = 1.8 Hz, 1H), 7.6 – 7.5 (m, 1H), 7.4 – 7.2 (m, 27H), 5.3 – 5.2 (m, 2H), 5.1 – 4.9 (m, 9H), 4.8 – 4.7 (m, 3H), 4.6 – 4.5 (m, 2H), 4.4 – 4.3 (m, 4H), 4.16 (d, *J* = 9.6 Hz, 1H), 4.0 – 3.9 (m, 2H), 3.6 – 3.5 (m, 1H), 3.4 – 3.2 (m, 6H), 3.17 (s, 3H), 1.8 – 1.7 (m, 2H), 1.4 – 1.3 (m, 2H), 1.3 – 1.2 (m, 24H), 0.86 (t, *J* = 6.6 Hz, 3H); ^{31}P NMR ($CDCl_3$, 162 MHz) δ -0.59, -6.71, -6.94; ESI-HRMS for $[M+H]^+$ $C_{70}H_{94}O_{20}P_3$: calcd 1347.5551, found 1347.5527.

Compound 5

YL2035-092-1

Item #

Batch #

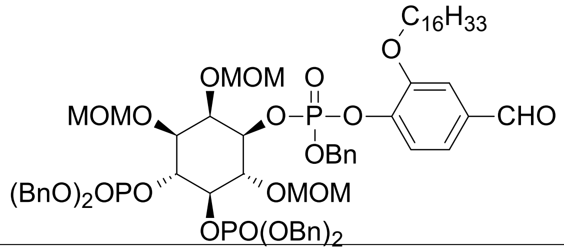

File Name	\\sulfur\private\nmrdata\JEOL_2021\YL-2035-092-1\YL-2035-092-1_PROTON_11-Feb-2021-1-1.jdf				
Date	11 Feb 2021 14:21:20	***Nucleus***	1H	***Frequency (MHz)***	399.7822
Solvent	CHLOROFORM-d	***Number of Transients***	16	***Origin***	JEOL ECZ400S Ca
Temperature (degree C)	20.700				

^{1}H NMR (CHLOROFORM-d, 400 MHz) δ 9.89 (d, 1H, *J*=1.8 Hz), 7.5-7.6 (m, 1H), 7.2-7.4 (m, 27H), 5.2-5.3 (m, 2H), 4.9-5.1 (m, 9H), 4.7-4.8 (m, 3H), 4.5-4.6 (m, 2H), 4.3-4.4 (m, 4H), 4.16 (d, 1H, *J*=9.6 Hz), 3.9-4.0 (m, 2H), 3.5-3.6 (m, 1H), 3.2-3.4 (m, 6H), 3.17 (s, 3H), 1.7-1.8 (m, 2H), 1.3-1.4 (m, 2H), 1.2-1.3 (m, 24H), 0.86 (t, 3H, *J*=6.6 Hz)

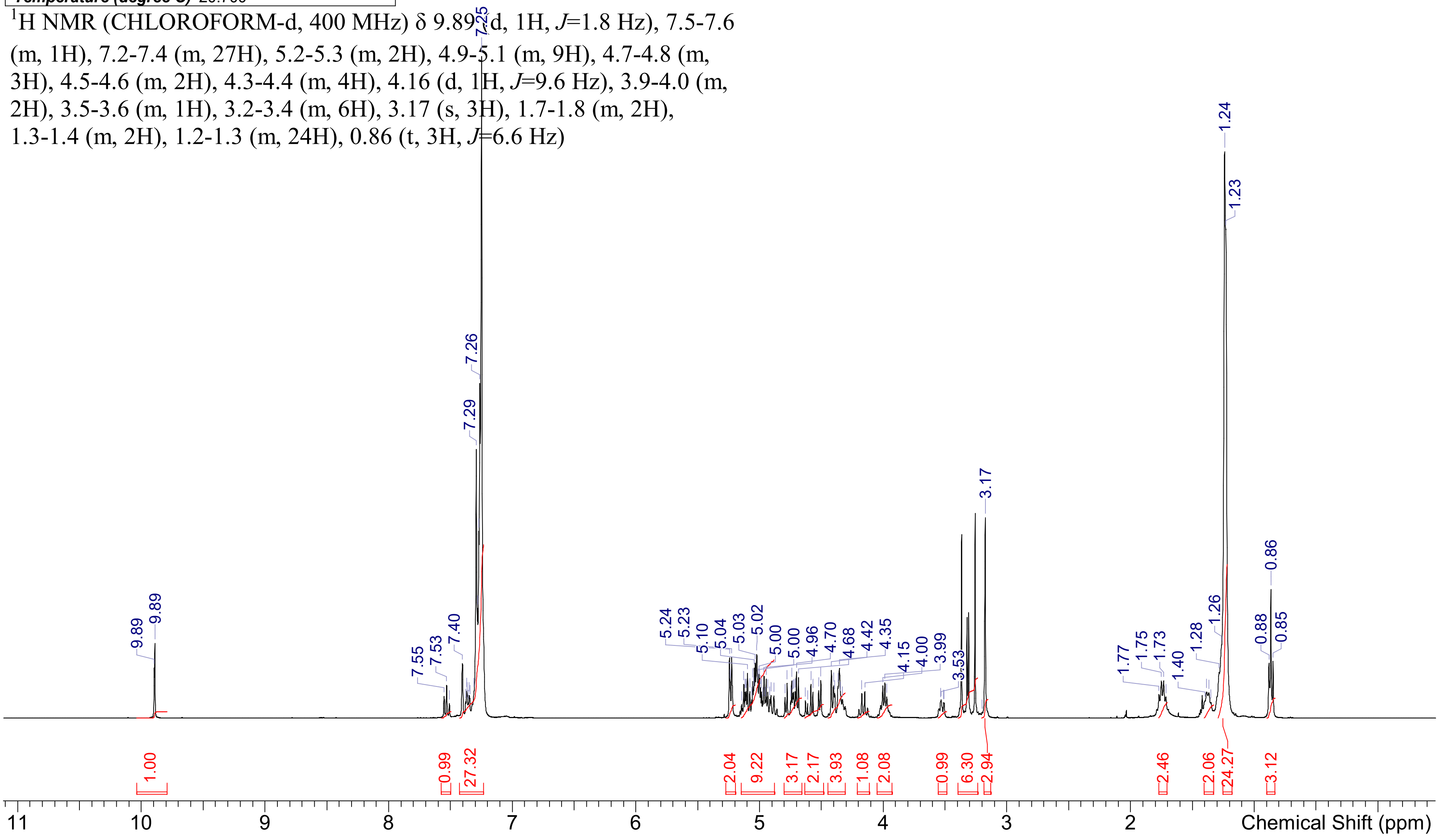

Compound 5

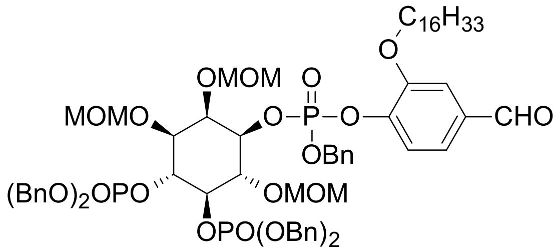

YL2034-092-1

Item #

Batch #

File Name	\\sulfur\private\nmrdata\JEOL_2021\YL-2035-092-1\YL-2035-092-1_PHOSPHORUS_11-Feb-2021-1-1.jdf				
Date	11 Feb 2021 14:24:12	***Nucleus***	31P	***Frequency (MHz)***	161.8347
Solvent	CHLOROFORM-d	***Number of Transients***	16	***Origin***	JEOL ECZ400S Ca
Temperature (degree C)	20.700				

^{31}P NMR (CHLOROFORM-d, 162 MHz) δ -0.8--0.3 (m, 1P), -7.2--6.6 (m, 1P)

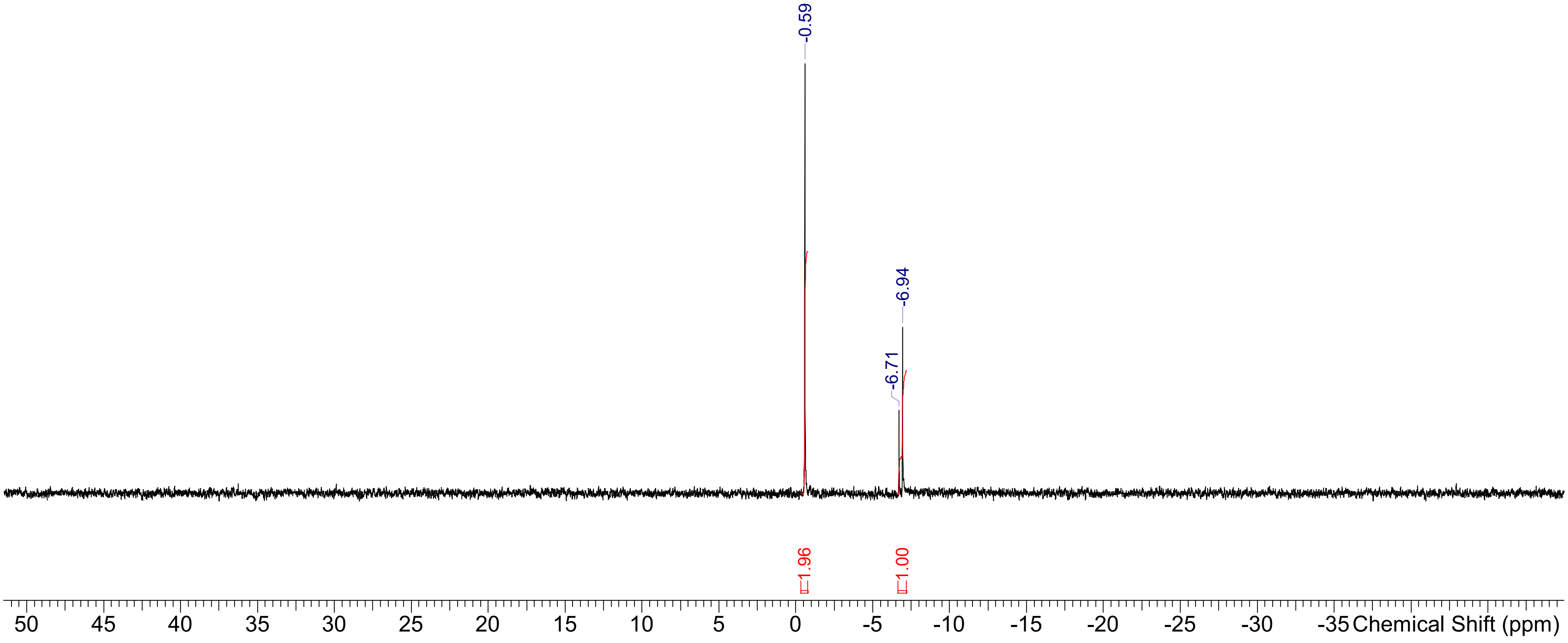

Intermediate 6. $NaBH_4$ (326 mg, 8.62 mmol, 2.2 eq) was added portion-wise to a solution of **5** (5.32 g, 3.95 mmol, 1 eq) in THF (50 mL) and the reaction stirred at rt for 4 h. The reaction was then filtered and concentrated to give an oily residue. The residue was purified by flash LC on a silica gel column, eluting with 80:20 to 50:50 hexanes:acetone gradient, to provide **6** (3.84 g, 72%) as a colorless oil. TLC R_f = 0.45 (50:50 hexanes:acetone); ^1H NMR ($CDCl_3$, 400 MHz) δ 7.4 – 7.2 (m, 26H), 6.94 (s, 1H), 6.81 (dd, *J* = 1.8, 8.3 Hz, 1H), 5.3 – 5.2 (m, 2H), 5.2 – 4.9 (m, 9H), 4.7 – 4.6 (m, 6H), 4.5 – 4.5 (m, 1H), 4.4 – 4.3 (m, 3H), 4.23 (s, 1H), 4.2 – 4.1 (m, 1H), 4.0 – 3.9 (m, 2H), 3.5 – 3.4 (m, 1H), 3.4 – 3.3 (m, 4H), 3.26 (s, 2H), 3.17 (s, 3H), 2.2 – 1.9 (m, 1H), 1.72 (sxt, *J* = 7.3 Hz, 2H), 1.4 – 1.2 (m, 26H), 0.87 (t, *J* = 6.6 Hz, 3H); ^{31}P NMR ($CDCl_3$, 162 MHz) δ -0.61, -6.01, -6.16; ESI-HRMS for $[M+H]^+$ $C_{70}H_{96}O_{20}P_3$: calcd 1349.5708, found 1349.5718.

Compound 6

WH15 Compd 15 C16

Item #

Batch #

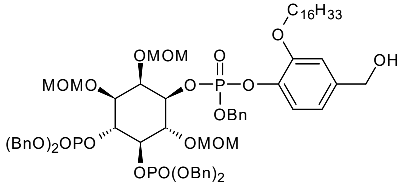

File Name	\\sulfur\private\nmrdata\JEOL_2021\KS-1987-136\KS-1987-136_PROTON_09-Feb-2021-1-1.jdf				
Date	09 Feb 2021 09:05:27	***Nucleus***	1H	***Frequency (MHz)***	399.7822
Solvent	CHLOROFORM-d	***Number of Transients***	16	***Origin***	JEOL ECZ400S Ca
Temperature (degree C)	20.500				

^{1}H NMR (CHLOROFORM-d, 400 MHz) δ 7.2-7.4 (m, 26H), 6.94 (s, 1H), 6.81 (dd, 1H, *J*=1.8, 8.3 Hz), 5.2-5.3 (m, 2H), 4.9-5.2 (m, 9H), 4.6-4.7 (m, 6H), 4.5-4.5 (m, 1H), 4.3-4.4 (m, 3H), 4.23 (s, 1H), 4.1-4.2 (m, 1H), 3.9-4.0 (m, 2H), 3.4-3.5 (m, 1H), 3.3-3.4 (m, 4H), 3.26 (s, 2H), 3.17 (s, 3H), 1.9-2.2 (m, 1H), 1.72 (sxt, 2H, *J*=7.3 Hz), 1.2-1.4 (m, 26H), 0.87 (t, 3H, *J*=6.6 Hz)

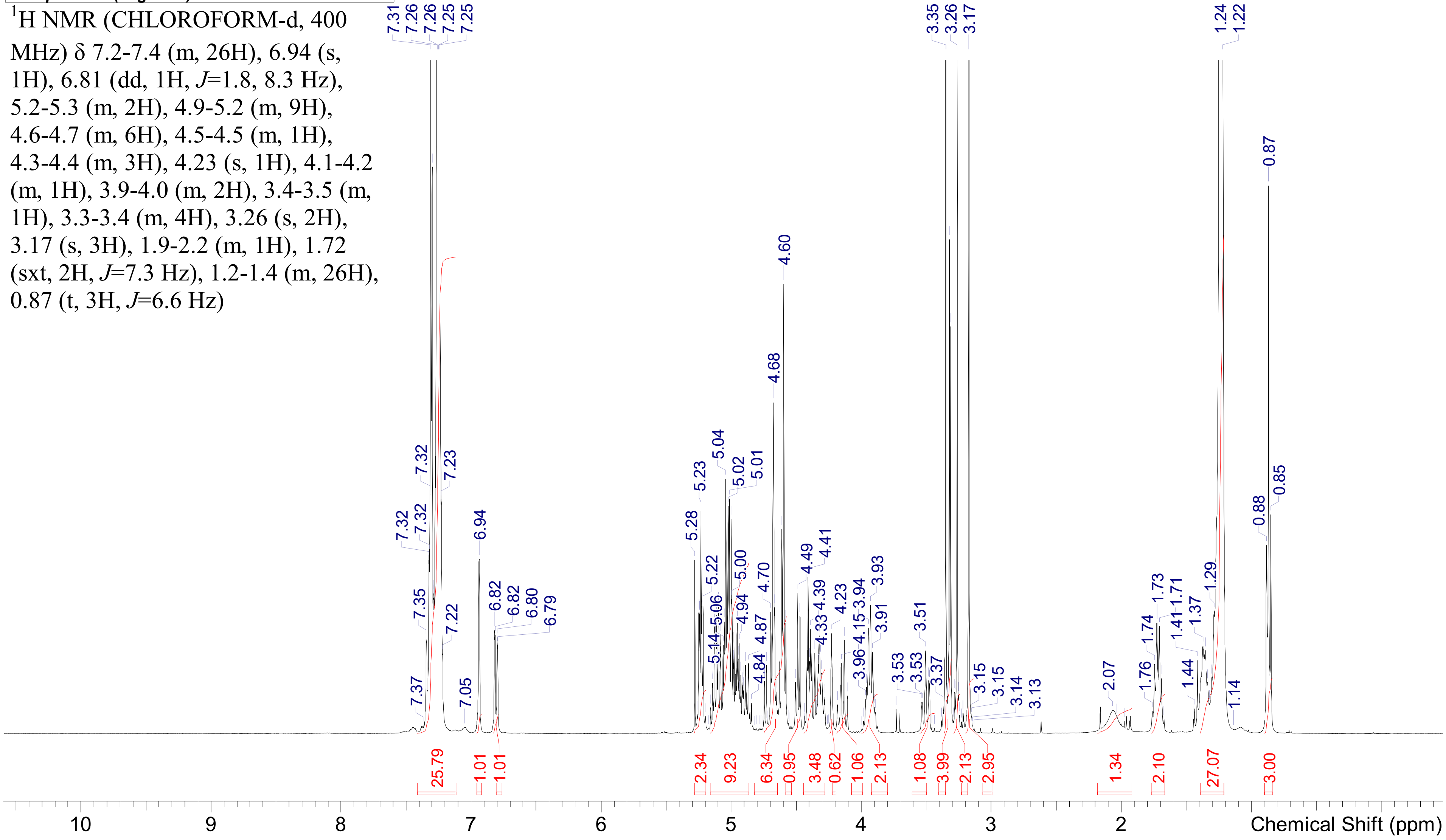

Compound 6

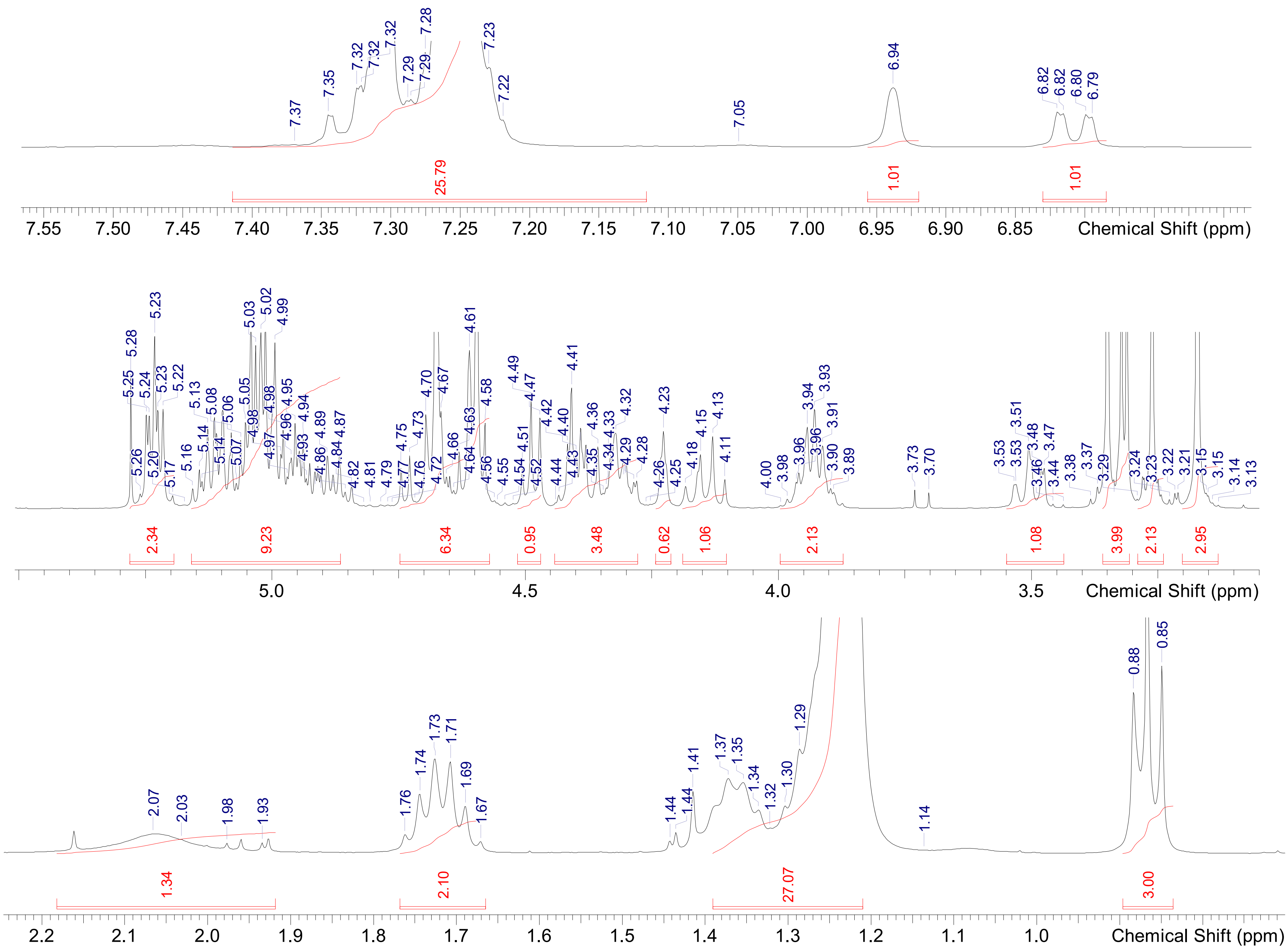

Compound 6

WH15 Compd 15 C16

Item #

Batch #

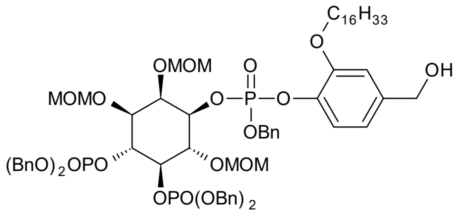

File Name	\\sulfur\private\nmrdata\JEOL_2021\KS-1987-136\KS-1987-136_PHOSPHORUS_09-Feb-2021-1-1.jdf				
Date	09 Feb 2021 09:21:58	***Nucleus***	31P	***Frequency (MHz)***	161.8347
Solvent	CHLOROFORM-d	***Number of Transients***	16	***Origin***	JEOL ECZ400S Ca
Temperature (degree C)	20.500				

^{31}P NMR (CHLOROFORM-d, 162 MHz) δ -0.61 (s, 1P), -0.63 (s, 1P), -6.01 (s, 1P), -6.16 (s, 1P)

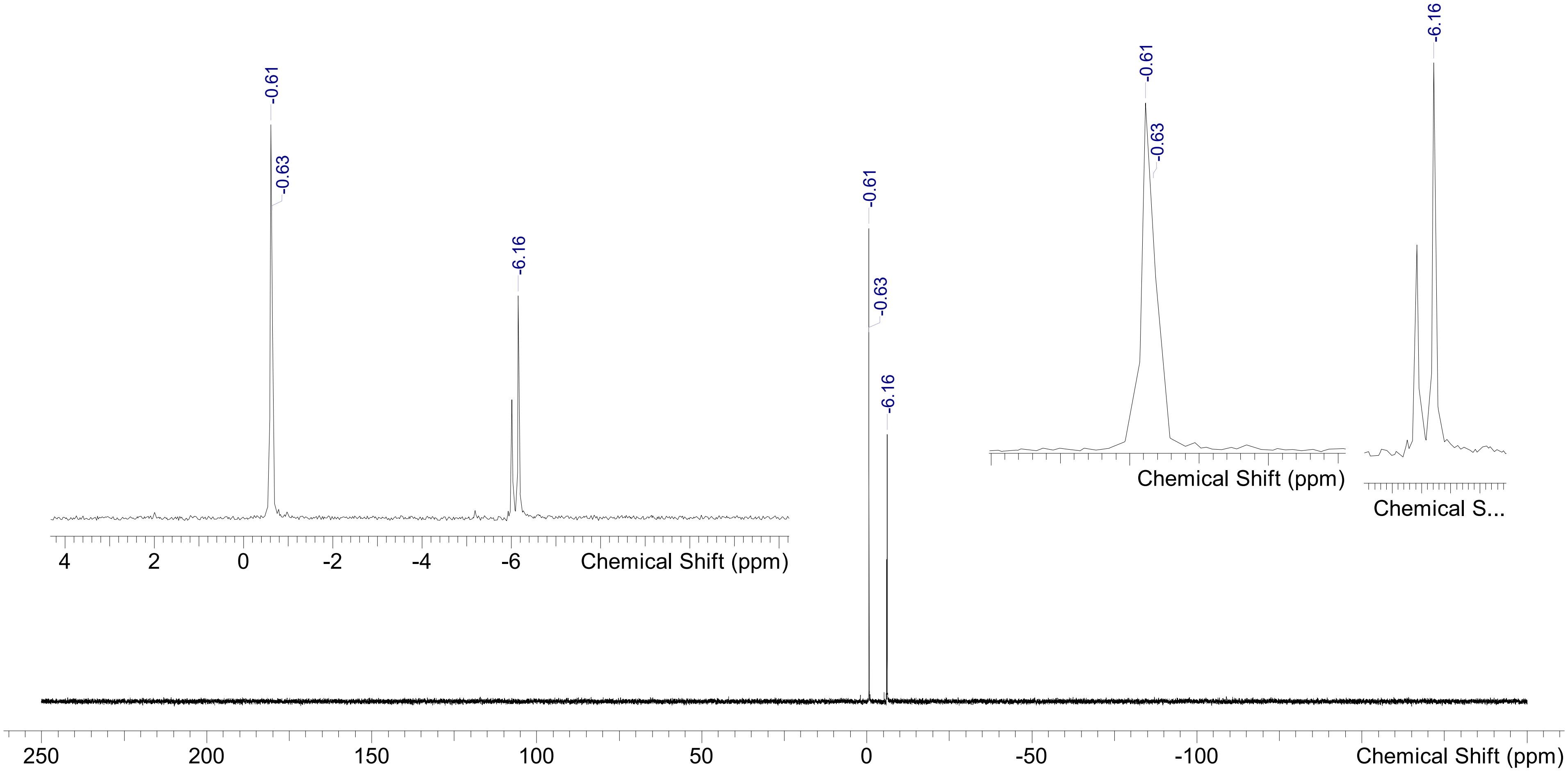

Compound 6

WH15 Compd 15 C16 **50 degree**

Item #

Batch #

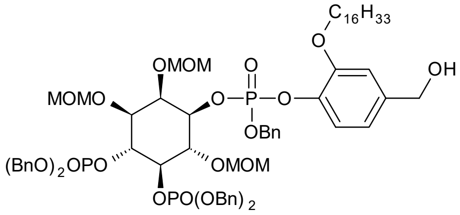

File Name	\\sulfur\private\nmrdata\JEOL_2021\KS-1987-136\KS-1987-136_PHOSPHORUS_09-Feb-2021-2-1.jdf				
Date	09 Feb 2021 09:36:26	***Nucleus***	31P	***Frequency (MHz)***	161.8347
Solvent	CHLOROFORM-d	***Number of Transients***	16	***Origin***	JEOL ECZ400S Ca
Temperature (degree C)	50.000				

^{31}P NMR (CHLOROFORM-d, 162 MHz) δ -0.73 (s, 1P), -6.15 (s, 1P)

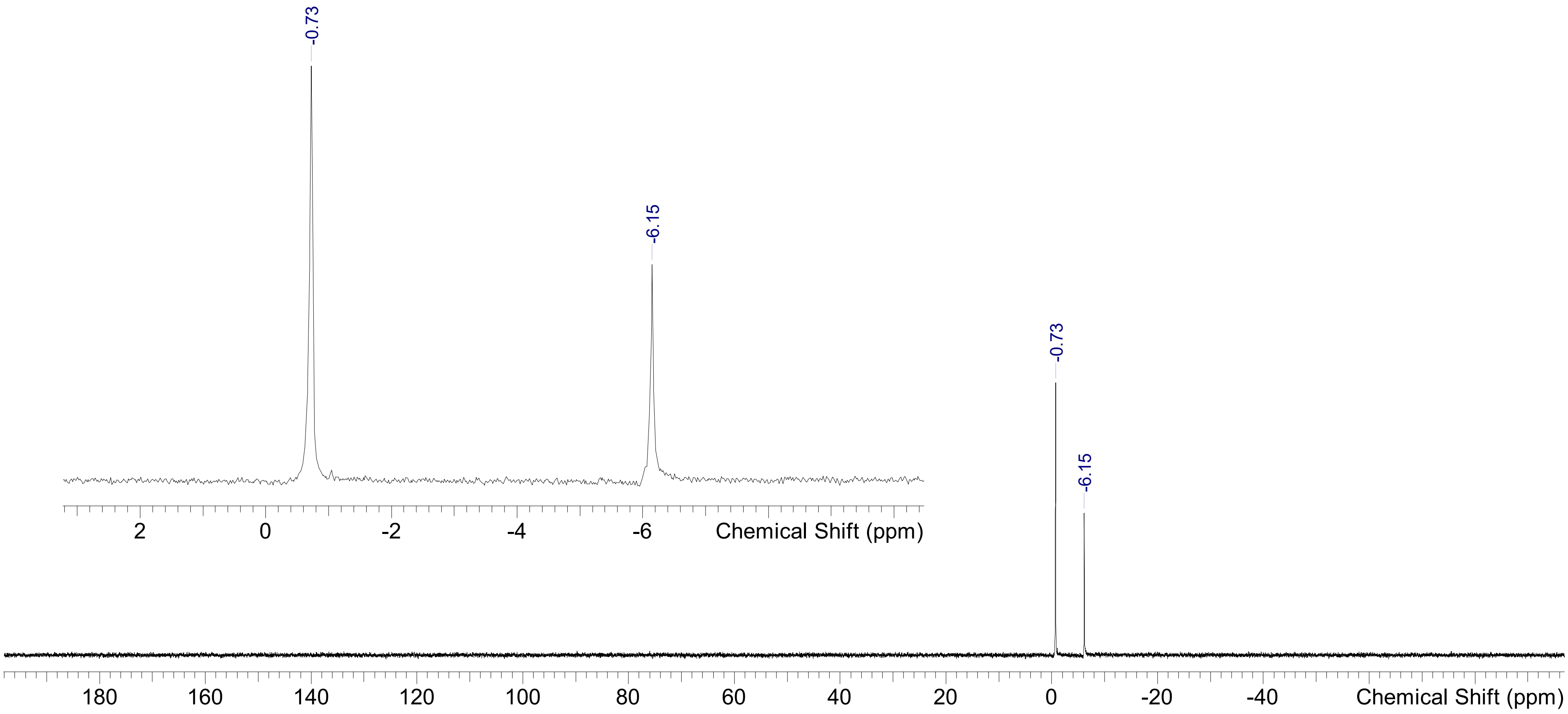

Intermediate 9. Preparation of isocyanate **8** followed a modified version of a procedure from Kim et al. (Bioorganic Chemistry, 2020, 94, art. no. 103405). 7-Amino-4-trifluoromethyl-coumarin (**7**, 58 mg, 0.253 mmol, 1.9 eq) was suspended in anhydrous dichloroethane (5 mL). NEt_3 (69 µL, 0.495 mmol, 3.6 eq) was added and the mixture was stirred at rt for ~15 min. Triphosgene (73 mg, 0.246 mmol, 1.8 eq) was then added. The reaction mixture was heated to 90 °C for 1 hr and became a solution. Complete conversion of **7** to **8** was confirmed by quenching an aliquot with MeOH to form the methyl carbamate of **7** and then checking TLC or LC-MS. The reaction mixture was cooled to rt and concentrated in vacuo to yield crude **8** as a yellowish white semisolid.

To crude **8** was added anhydrous DCM (1 mL) and stirring was initiated to produce a yellow solution. Activated 4 Å molecular sieves were added, followed by dropwise addition of a solution of intermediate **6** (199 mg, 92.46% purity, 0.136 mmol, 1 eq) in anhydrous DCM (1 mL), and finally a rinse of anhydrous DCM (1 mL) from the vial that contained **6**. NEt_3 (69 µL, 0.495 mmol, 3.6 eq) was then added, followed by DMAP (3 mg, 0.025 mmol, 0.2 eq). The bright yellow solution was allowed to stir at rt for ~1.5 h with TLC or LC-MS monitoring. When **6** was no longer detected, the reaction mixture was quenched by addition of aqueous half-saturated NH_4Cl solution (5 mL). The organic layer was set aside and the aqueous solution was extracted with DCM (2 x 5 mL). The combined organic layers were dried over Na_2SO_4 and concentrated in vacuo. The residue was purified the same day by flash LC on a 12-g silica gel column, eluting with 90:10 to 60:40 hexanes:acetone gradient to provide **9** (209 mg, 96%) as a barely yellow white solid. TLC R_f = 0.55 (50:50 hexanes:acetone); ^{1}H NMR (DMSO-d_6, 500 MHz) δ 10.47 (s, 1H), 7.70 – 7.63 (m, 2H), 7.50 (dd, *J* = 8.9, 2.1 Hz, 1H), 7.38 – 7.20 (m, 27H), 7.00 (dd, *J* = 8.2, 2.0 Hz, 1H), 6.87 (s, 1H), 5.29 – 5.20 (m, 2H), 5.17 (s, 2H), 5.13 – 5.05 (m, 2H), 5.04 – 4.96 (m, 6H), 4.85 – 4.49 (m, 8H), 4.43 (d, *J* = 7.2 Hz, 1H), 4.33 – 4.27 (m, 1H), 4.04 – 3.95 (m, 4H), 3.28 – 3.22 (m, 2H), 3.21 – 3.15 (m, 5H), 1.65 (p, *J* = 7.2 Hz, 2H), 1.38 – 1.29 (m, 2H), 1.28 – 1.10 (m, 25H), 0.84 (t, *J* = 6.8 Hz, 3H) [Note: one signal at 3.31 ppm, accounting for 1H, is mostly obscured by H_2O and is not reported in this list]; QTOF-HRMS for $[M+H]^+$ $C_{81}H_{100}F_3NO_{23}P_3$: calcd 1604.5849, found 1604.5845

Compound 9

putt-massey-500-1.493.fid
H1 standard parameters, BBFO probe. DEB-3-044-C in DMSO-d6, 299K. Pos. 43.

^{1}H NMR (500 MHz, DMSO) δ 10.47 (s, 1H), 7.70 – 7.63 (m, 2H), 7.50 (dd, J = 8.9, 2.1 Hz, 1H), 7.38 – 7.20 (m, 27H), 7.00 (dd, J = 8.2, 2.0 Hz, 1H), 6.87 (s, 1H), 5.29 – 5.20 (m, 2H), 5.17 (s, 2H), 5.13 – 5.05 (m, 2H), 5.04 – 4.96 (m, 6H), 4.85 – 4.49 (m, 8H), 4.43 (d, J = 7.2 Hz, 1H), 4.33 – 4.27 (m, 1H), 4.04 – 3.95 (m, 4H), 3.28 – 3.22 (m, 2H), 3.21 – 3.15 (m, 5H), 1.65 (p, J = 7.2 Hz, 2H), 1.38 – 1.29 (m, 2H), 1.28 – 1.10 (m, 25H), 0.84 (t, J

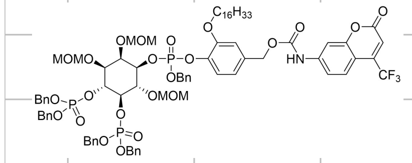

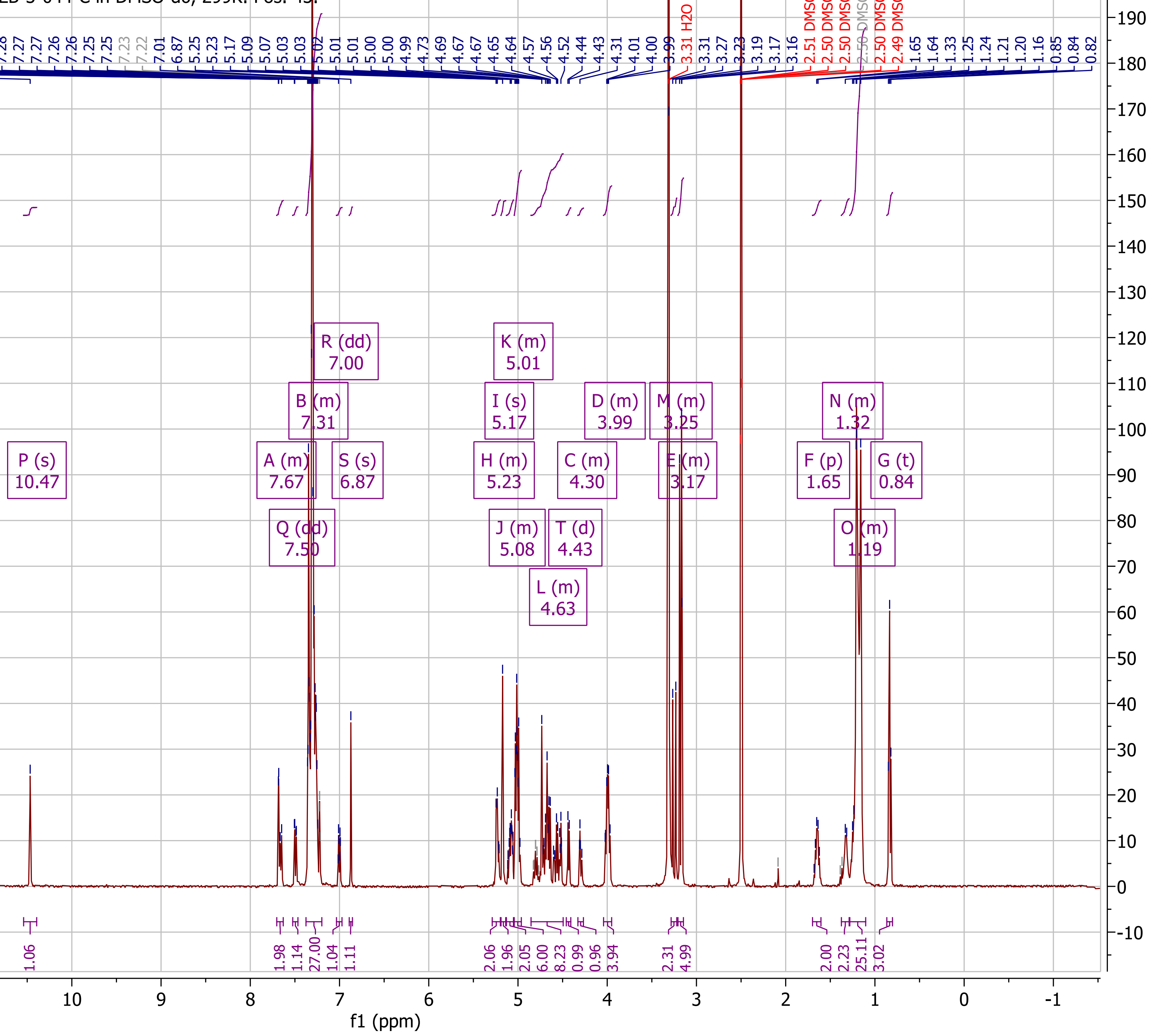

Compound 9

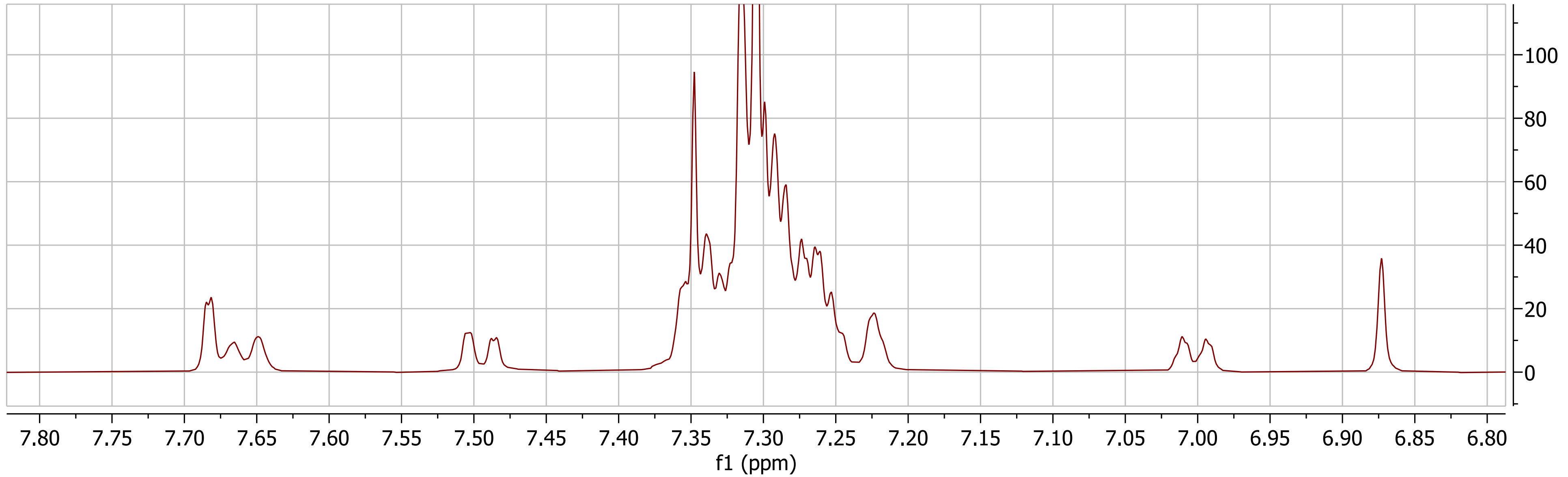

100
80
60
40
20
0
7.80 7.75 7.70 7.65 7.60 7.55 7.50 7.45 7.40 7.35 7.30 7.25 7.20 7.15 7.10 7.05 7.00 6.95 6.90 6.85 6.80
f1 (ppm)

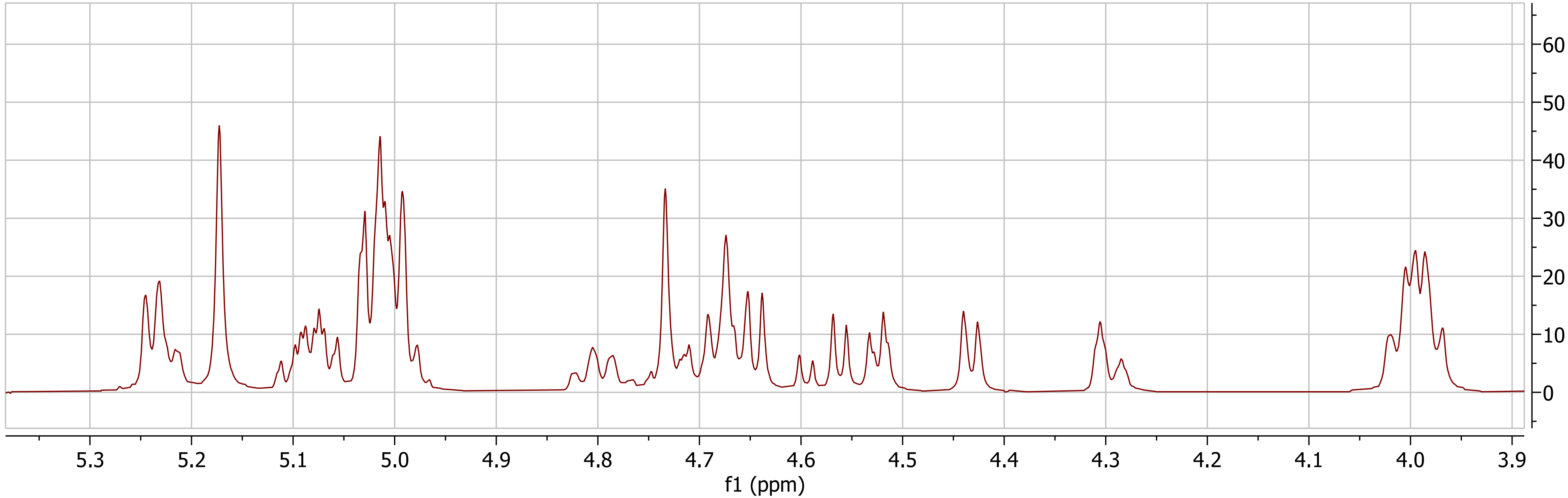

60
50
40
30
20
10
0
5.3 5.2 5.1 5.0 4.9 4.8 4.7 4.6 4.5 4.4 4.3 4.2 4.1 4.0 3.9
f1 (ppm)

Compound 9

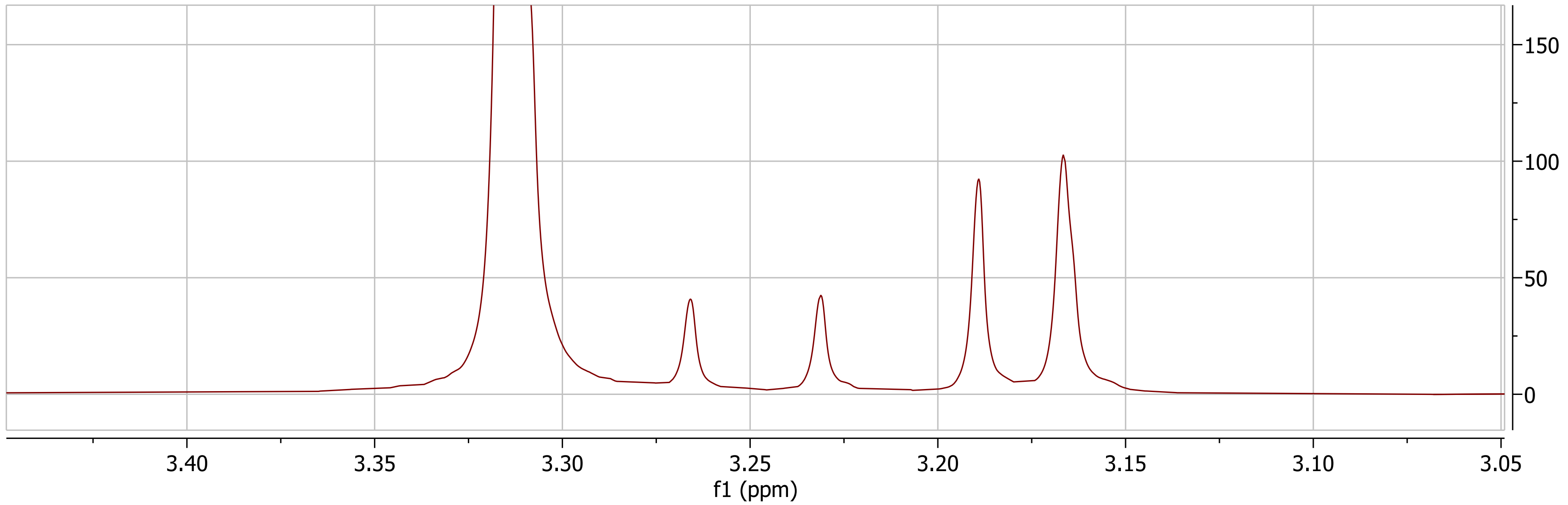
150
100
50
0
3.40
3.35
3.30
3.25
3.20
3.15
3.10
3.05
f1 (ppm)

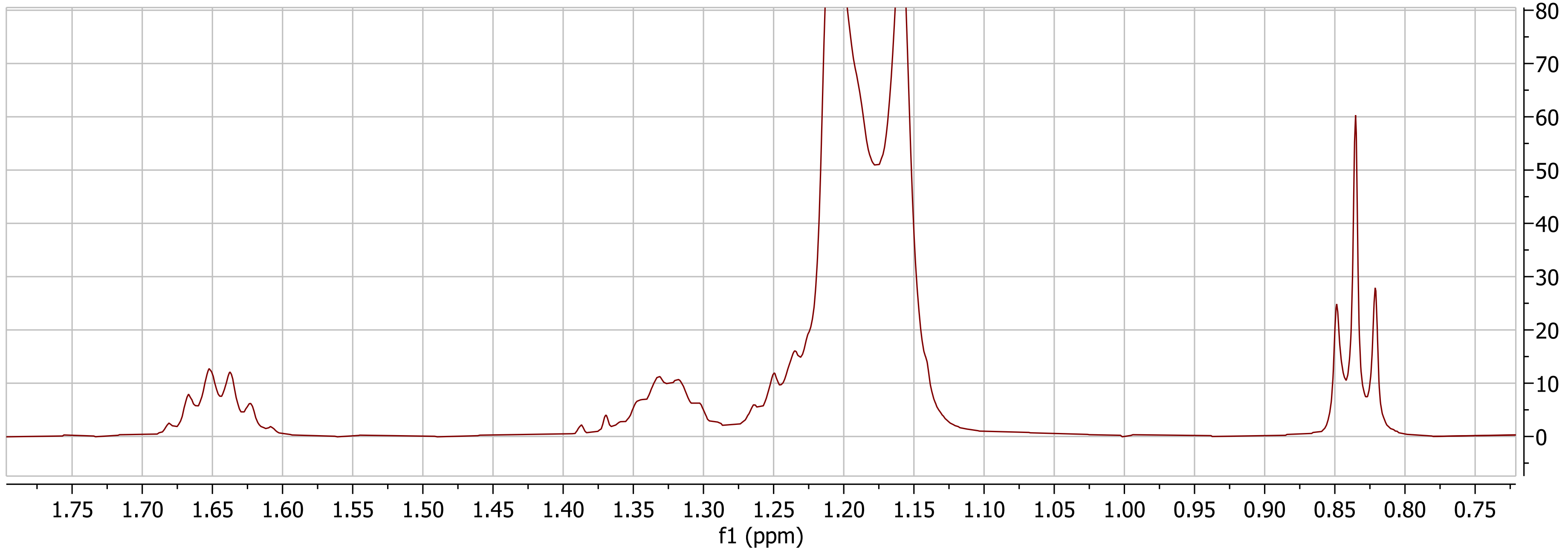
80
70
60
50
40
30
20
10
0
1.75
1.70
1.65
1.60
1.55
1.50
1.45
1.40
1.35
1.30
1.25
1.20
1.15
1.10
1.05
1.00
0.95
0.90
0.85
0.80
0.75
f1 (ppm)

C16CF3-coumarin. Intermediate **9** (209 mg, 0.130 mmol, 1 eq) was dissolved with stirring in anhydrous DCM (2 mL) to give a faint yellow solution. The solution was cooled to ~5 °C in an ice-water bath, and then freshly distilled TMSBr (2.0 mL, 15 mmol, 120 eq) was added dropwise. The mixture was stirred for 30 min in the ice-water bath, and then it was removed from the bath and allowed to warm to rt for 1 h 20 min. The reaction progress was followed by LC-MS and it showed complete disappearance of intermediate **9**. The reaction mixture appeared as a yellow solution at this time. The mixture was concentrated in vacuo and the resulting yellow oil was dried under high vacuum for 1 hr.

To the residue was added MeOH (2 mL), and the resulting solution was stirred at rt for 1 h. The solution was concentrated in vacuo and the resulting yellow oil was dried under high-vacuum for 1 h. A portion (58 mg) of the crude product (145 mg) was dissolved by sequential addition of aqueous 1.0 M pH 8.5 TEAB buffer (0.2 mL), THF (0.4 mL), and MeOH (0.2 mL), thoroughly mixing after each addition, to give a clear yellow solution. In each of two separate runs, half of the obtained solution was subjected to flash LC on a 15.5-g C8-derivatized silica column, eluting with 75:25 to 50:50 to 0:100 H_2O:(50:50 THF:MeOH), to provide **C16CF3-coumarin** (48 mg, 70%) as a barely yellow white solid. [*Note: different preparations gave varying ratios of NEt_3 per substrate molecule; it was typically 3 ± 0.5 NEt_3 per substrate molecule. The NMR data below correspond to 3.0 NEt_3.*] TLC R_f = 0.00 (50:50 hexanes:acetone); ^{1}H NMR (CD_3OD, 500 MHz) δ 7.77 (d, *J* = 2.1 Hz, 1H), 7.65 (dd, *J* = 8.8, 2.0 Hz, 1H), 7.58 (d, *J* = 8.3 Hz, 1H), 7.40 (dd, *J* = 8.9, 2.2 Hz, 1H), 7.06 (s, 1H), 6.93 (dd, *J* = 8.2, 2.1 Hz, 1H), 6.72 (s, 1H), 5.16 (s, 2H), 4.47 – 4.27 (m, 2H), 4.25 – 4.10 (m, 1H), 4.08 – 3.98 (m, 4H), 3.69 – 3.53 (m, 1H), 3.17 (q, *J* = 7.3 Hz, 18H), 1.81 (p, *J* = 7.0 Hz, 2H), 1.51 – 1.42 (m, 2H), 1.39 – 1.18 (m, 51H), 0.89 (t, *J* = 6.8 Hz, 3H); ^{19}F NMR (CD_3OD, 470 MHz) δ -67.55; ^{31}P NMR (CD_3OD, 202 MHz) δ 3.36, 2.41, -4.05; QTOF-HRMS for $[M-H]^-$ $C_{40}H_{56}F_3NO_{20}P_3$, calcd 1020.2561, found 1020.2515.

putt-massey-500-1.931.fid
H1 standard parameters, BBFO probe. DEB-3-095-D in CD3OD, 299K. Pos. 31.
With mild presaturation of H2O at 4.85 ppm.

C16CF3-coumarin

^{1}H NMR (500 MHz, MeOD) δ 7.77 (d, *J* = 2.1 Hz, 1H), 7.65 (dd, *J* = 8.8, 2.0 Hz, 1H), 7.58 (d, *J* = 8.3 Hz, 1H), 7.40 (dd, *J* = 8.9, 2.2 Hz, 1H), 7.06 (s, 1H), 6.93 (dd, *J* = 8.2, 2.1 Hz, 1H), 6.72 (s, 1H), 5.16 (s, 2H), 4.47 – 4.27 (m, 2H), 4.25 – 4.10 (m, 1H), 4.08 – 3.98 (m, 4H), 3.69 – 3.53 (m, 1H), 3.17 (q, *J* = 7.3 Hz, 18H), 1.81 (p, *J* = 7.0 Hz, 2H), 1.51 – 1.42 (m, 2H), 1.39 – 1.18 (m, 51H), 0.89 (t, *J* = 6.8 Hz,

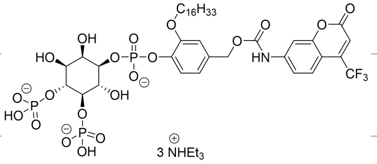

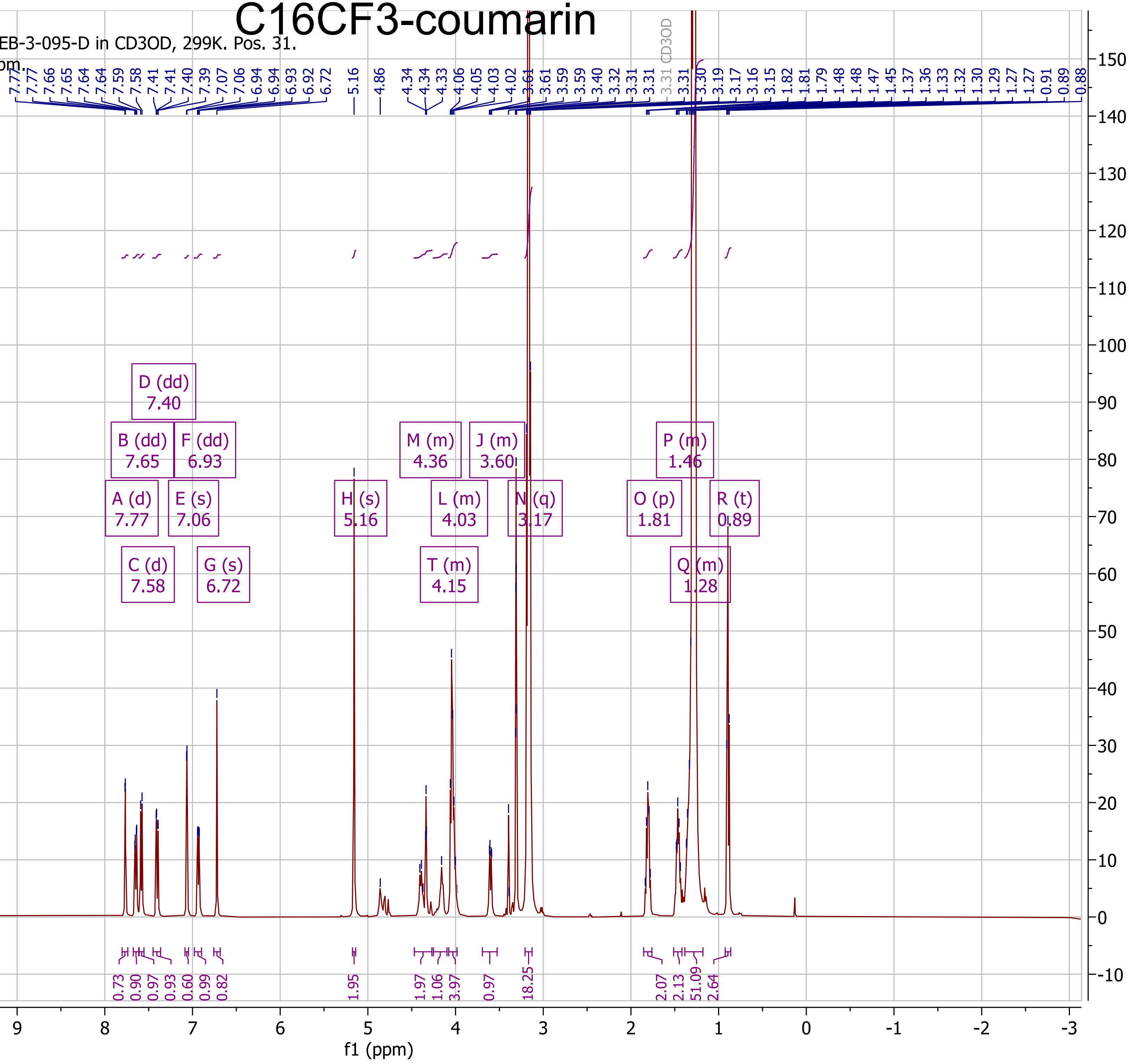

C16CF3-coumarin

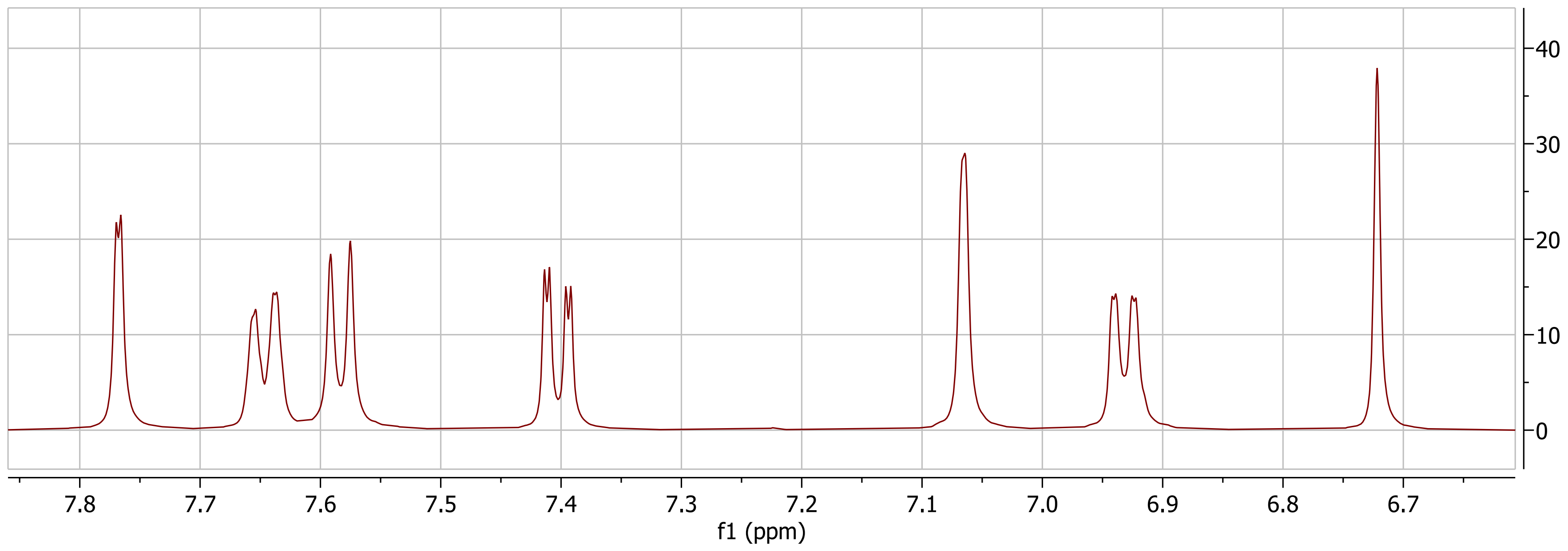

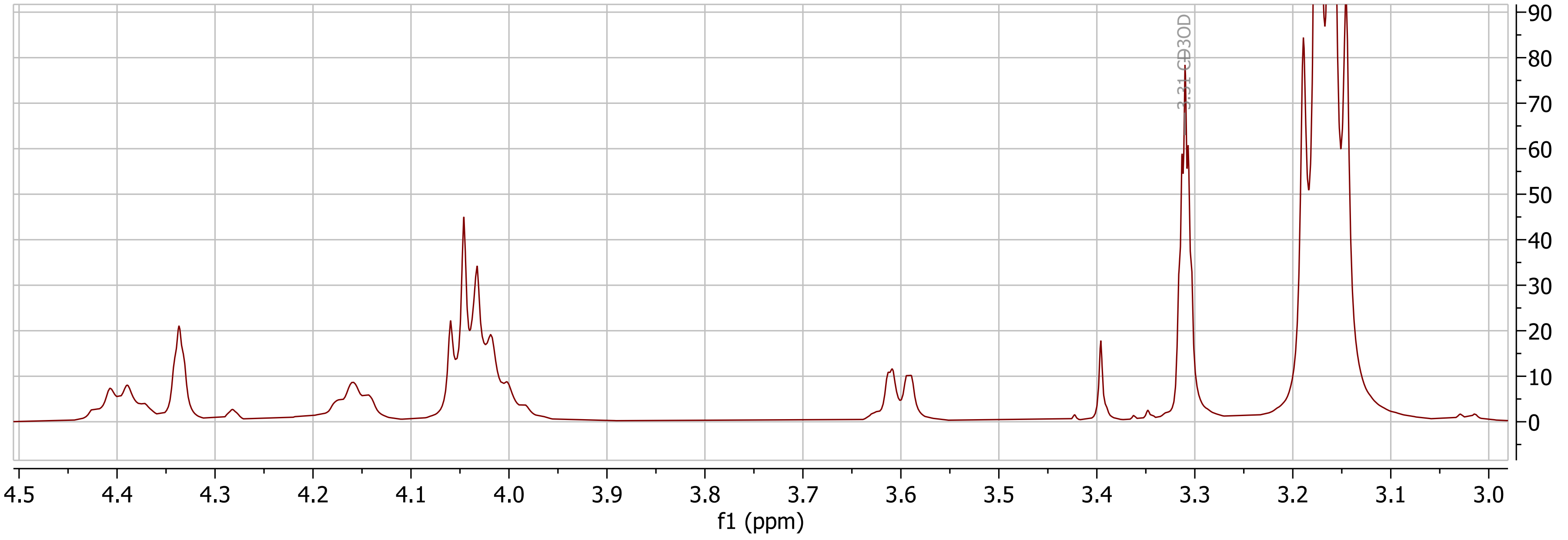

C16CF3-coumarin

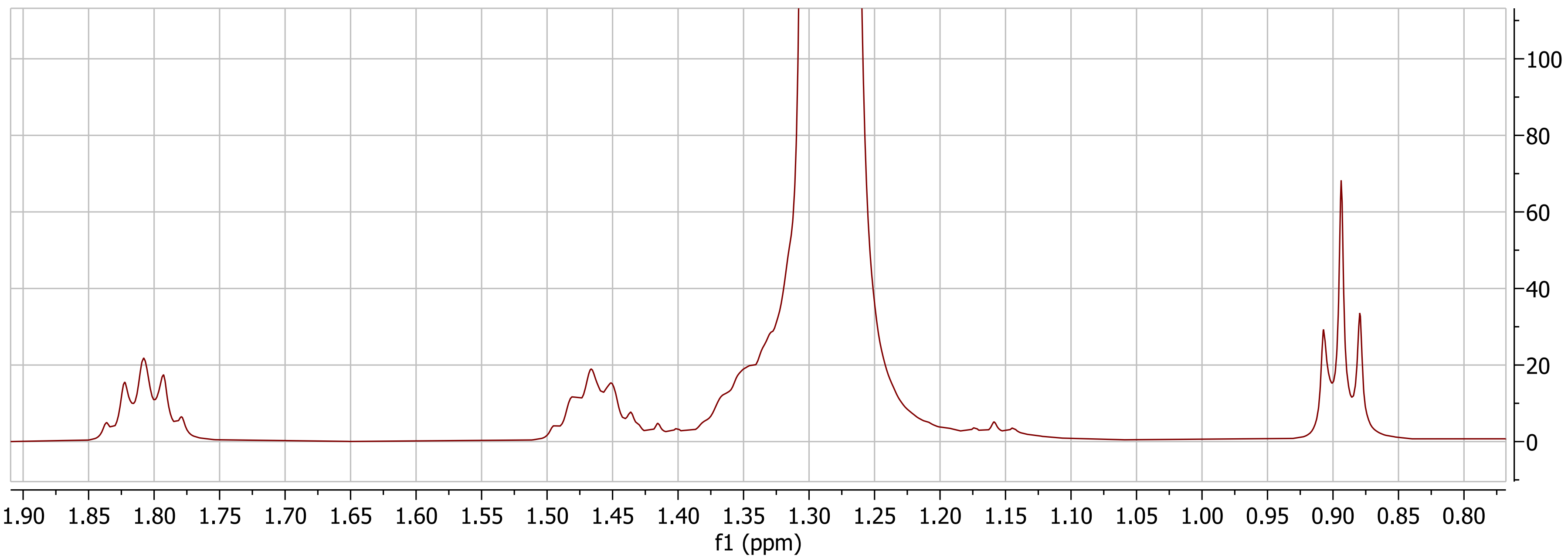

putt-massey-500-1.975.fid
F19 standard parameters, BBFO probe. DEB-3-095-D-2 in CD3OD, 299K. Pos. 34.

C16CF3-coumarin

^{19}F NMR (470 MHz, MeOD) δ -67.55.

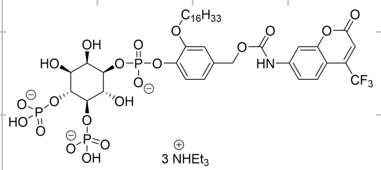

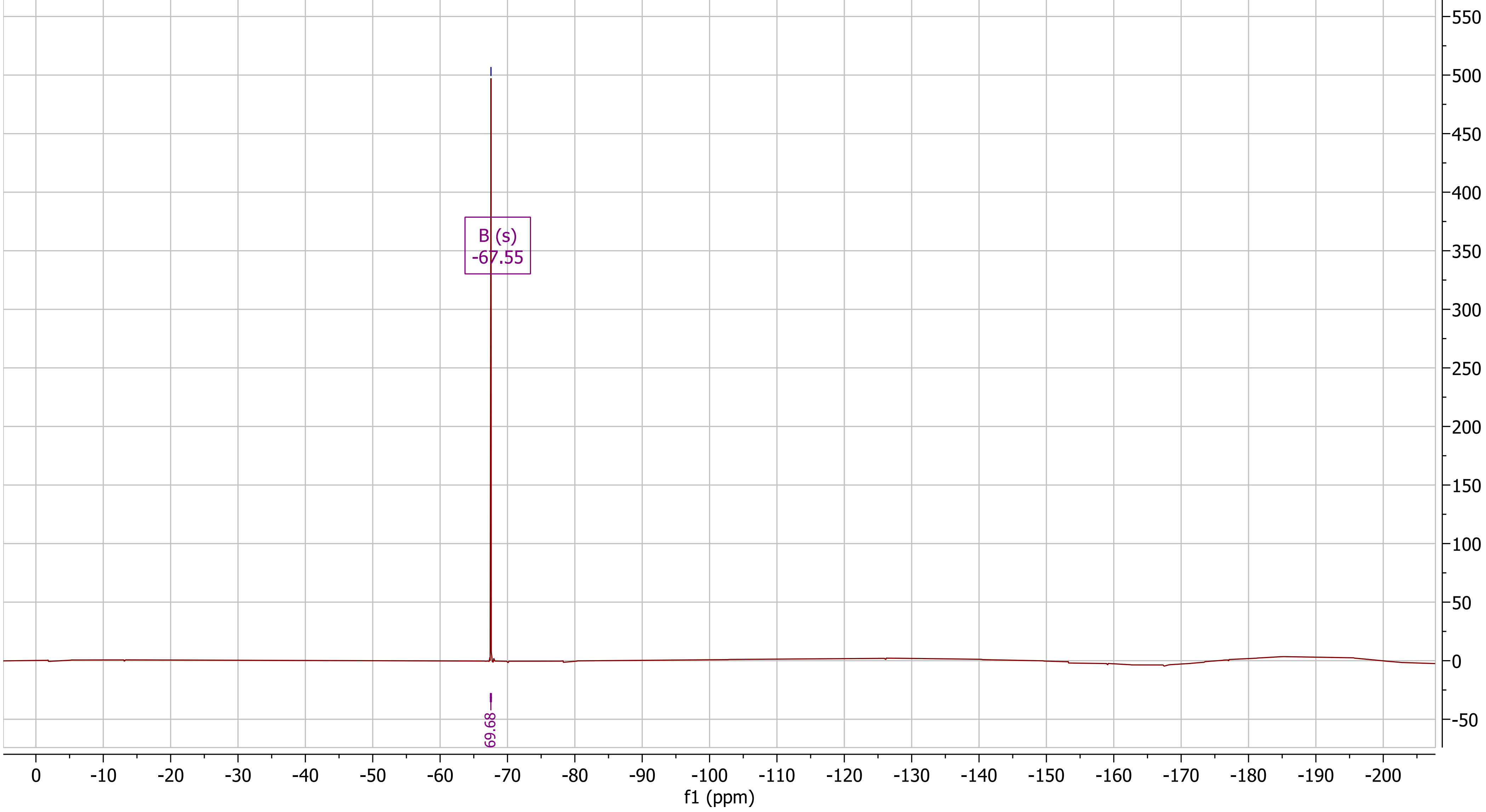

C16CF3-coumarin

putt-massey-500-1.976.fid
P31 standard parameters, BBFO probe. DEB-3-095-D-2 in CD3OD, 299K. Pos. 34.

^{31}P NMR (202 MHz, MeOD) δ 3.36, 2.41, -4.05.

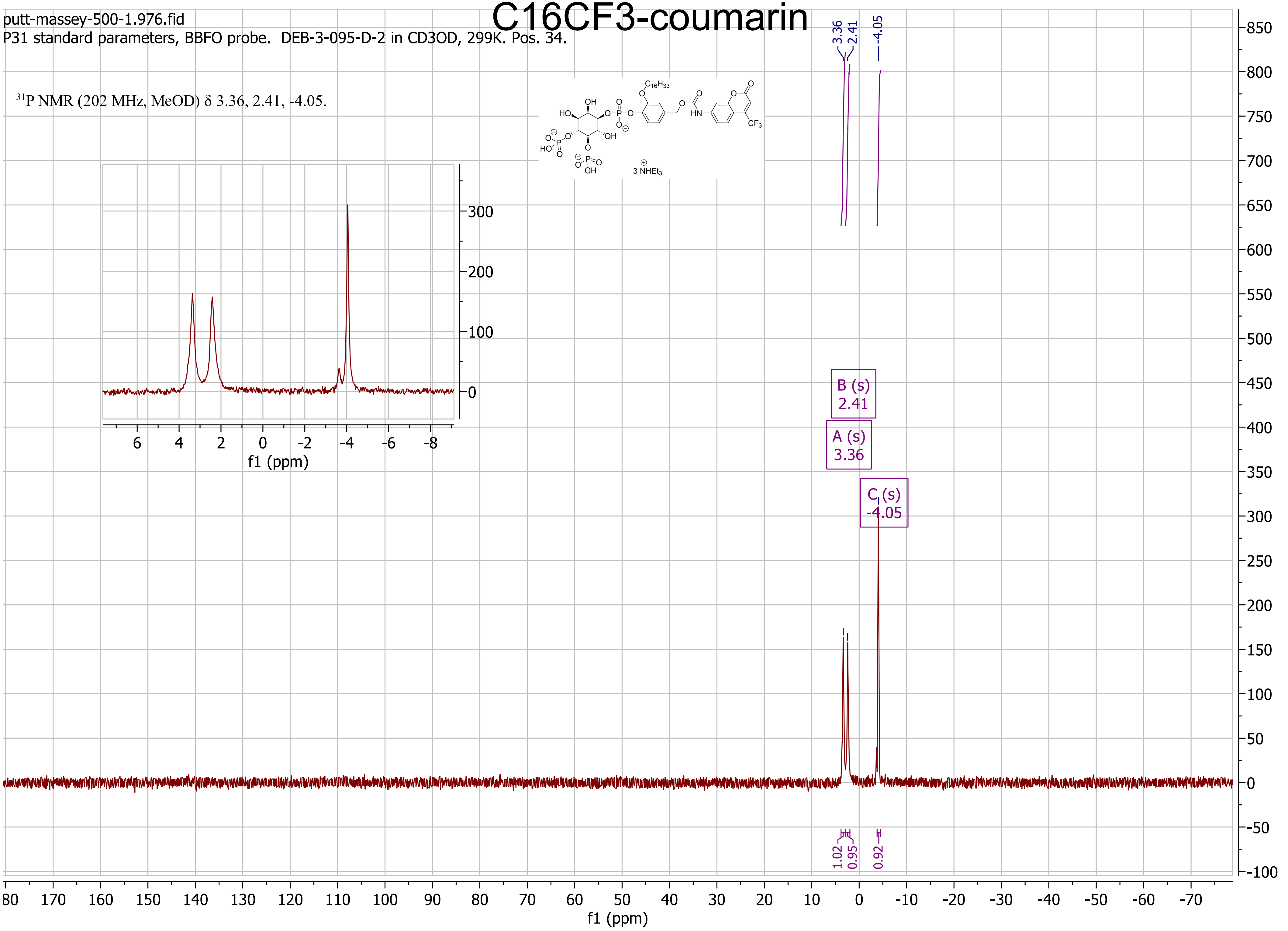

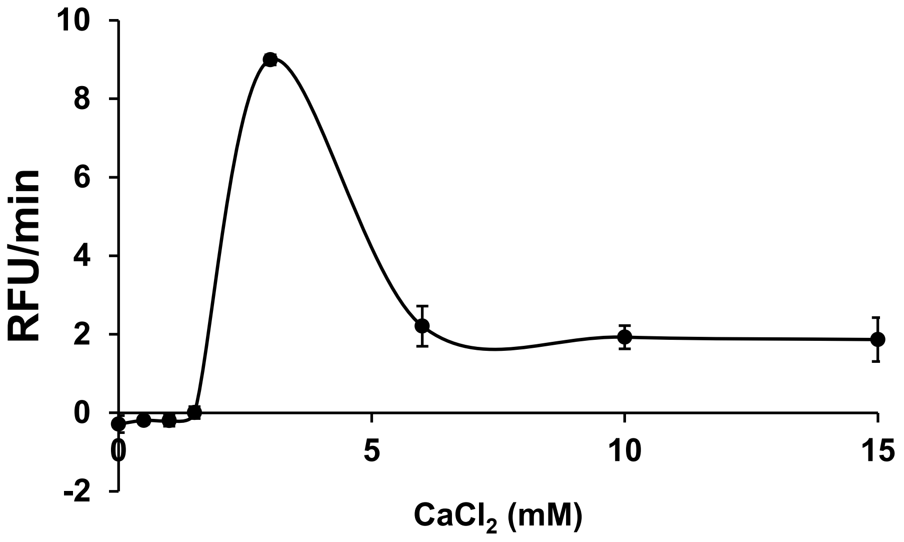

Supplementary Figure 1. **Calcium chloride optimizations for C16CF3-coumarin assay.** Effects of calcium chloride concentrations (0 – 15 mM) on enzymatic reactions of PLCγ2 (2.5 nM) and C16CF3-coumarin (5 μM).

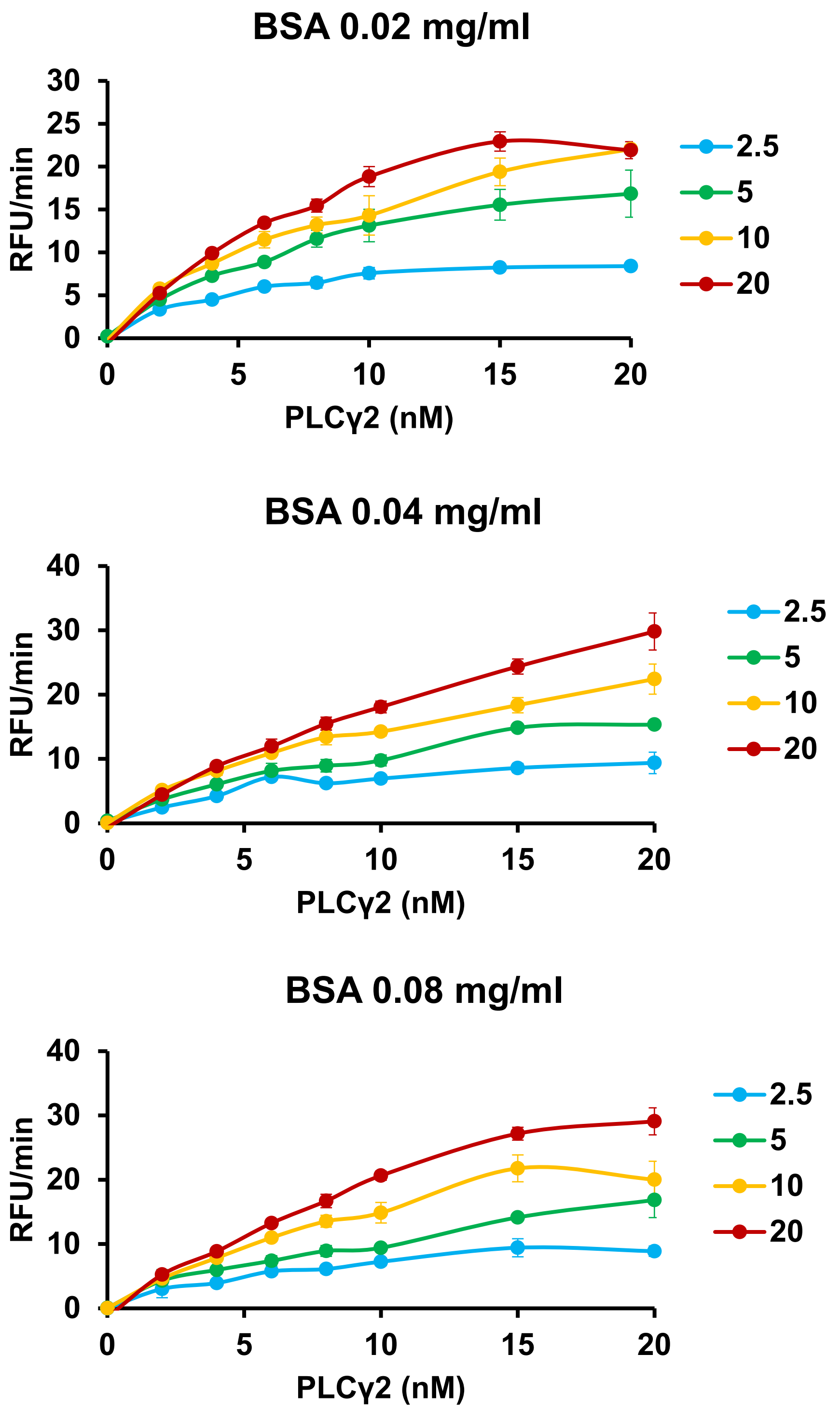

Supplementary Figure 2. BSA optimizations for C16CF3-coumarin assay. Effects of enzyme, substrate, and BSA concentrations on enzymatic reactions of PLCγ2 and C16CF3-coumarin [PLCγ2 (0 – 20 nM); C16CF3-coumarin (2.5 – 20 μM); BSA (0.02 – 0.08 mg/ml)]. The values are shown as the mean ± SD from three different experiments.

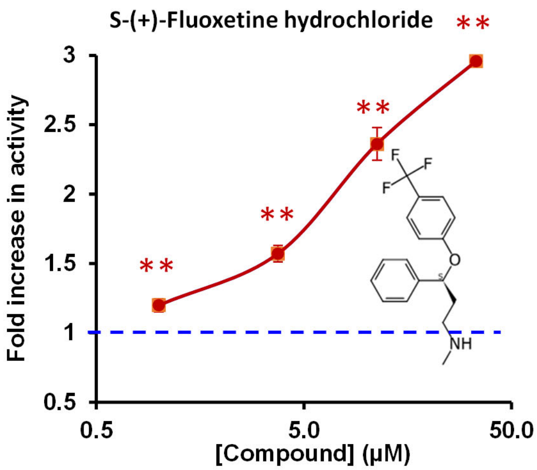

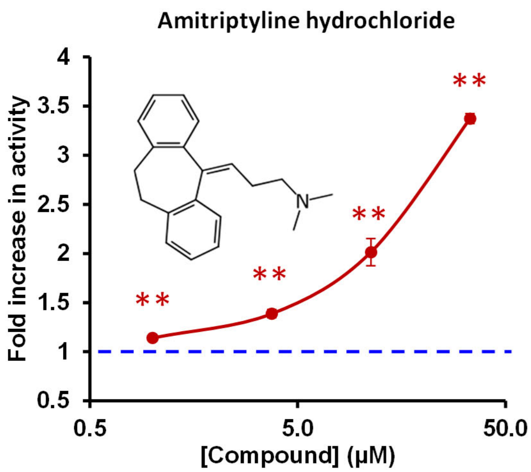

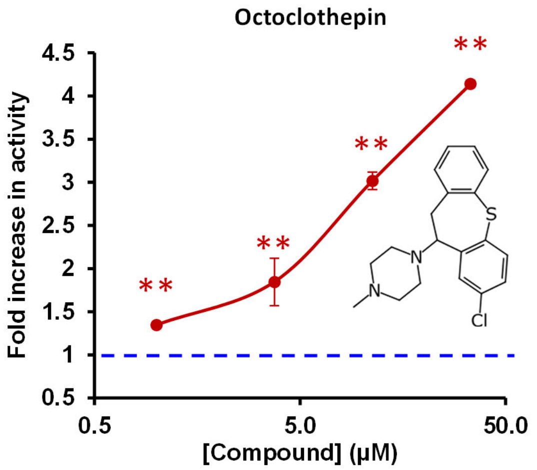

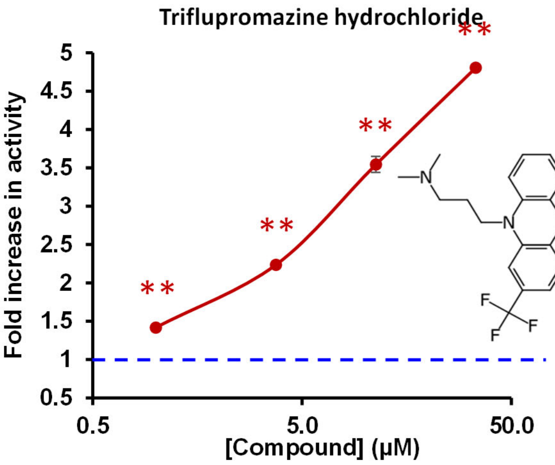

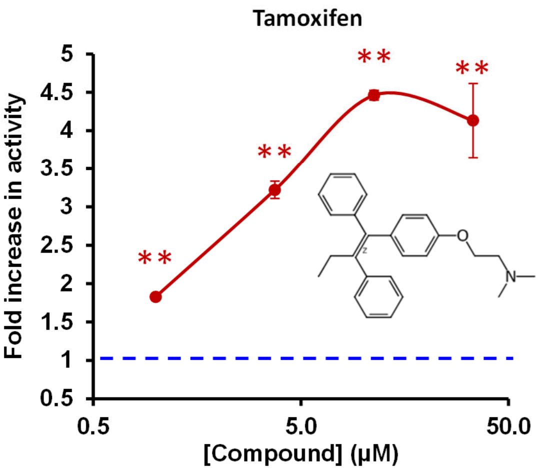

Supplementary Figure 3. Dose-response profiles of selected activator compounds from the LOPAC1280 screen. Various concentrations of the hit compounds (1 to 33 µM) were added to the wells of a 384-well plate along with 2.5 nM PLCγ2 and 5 µM C16CF3-coumarin. Fluorescence intensity was monitored every two minutes and the initial linear range (60 min) of the reaction profiles was calculated for the slope. The values are shown as the mean SD from three different experiments. Each experiment had triplicate wells. ***P* < 0.01, **P* < 0.05 represents significant differences versus the control group.

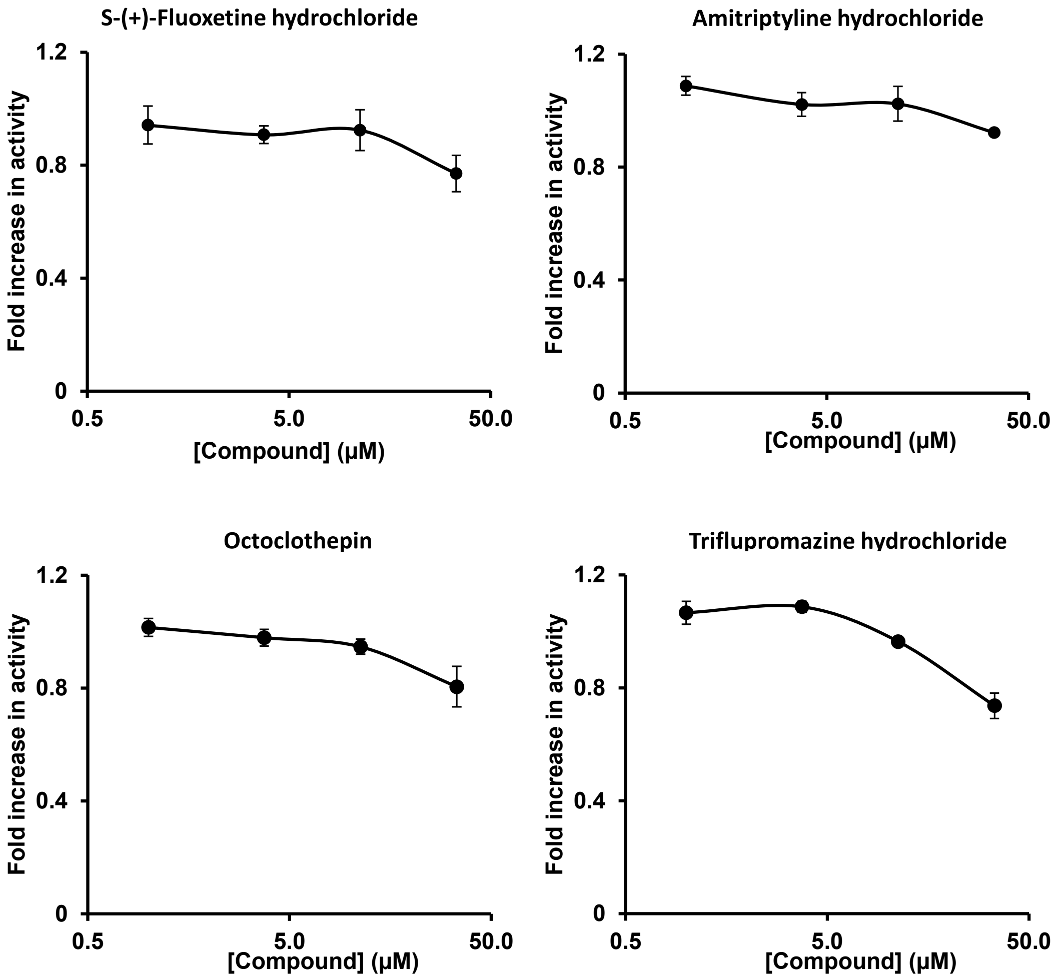

Supplementary Figure 4. Dose-response profiles of selected micelle activator compounds in the XY-69 assay. Various concentrations of the hit compounds (1 to 33 μM) were added to the wells of a 384-well plate along with 1 nM PLCγ2 and 2 μM XY-69 in liposomes. Fluorescence intensity was monitored every two minutes and the initial linear range (30 min) of the reaction profiles was used to calculate the slope. The values are shown as the mean SD from three different experiments. Each experiment had triplicate wells.

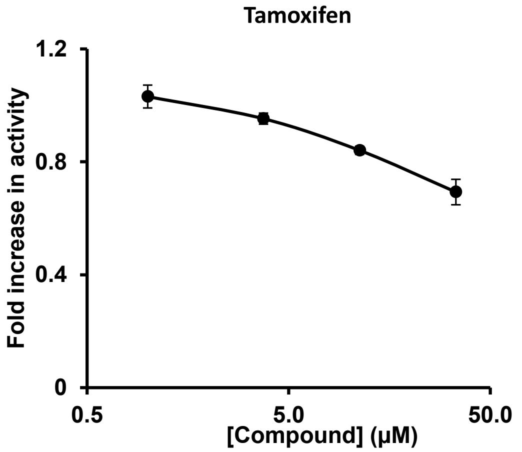

Supplement: S1 File — (PDF) [file pone.0299541.s001.pdf]
